# SoxB1 transcription factors are essential for initiating and maintaining neural plate border gene expression

**DOI:** 10.1242/dev.202693

**Published:** 2024-07-22

**Authors:** Elizabeth N. Schock, Joshua R. York, Austin P. Li, Ashlyn Y. Tu, Carole LaBonne

**Affiliations:** ^1^ Department of Molecular Biosciences, Northwestern University, Evanston, IL 60208, USA; ^2^ NSF-Simons National Institute for Theory and Mathematics in Biology, 875 N Michigan Avenue, Chicago, IL 60611, USA

**Keywords:** Neural plate border, Neural crest, SoxB1, Pou5f, *Xenopus*

## Abstract

SoxB1 transcription factors (Sox2/3) are well known for their role in early neural fate specification in the embryo, but little is known about functional roles for SoxB1 factors in non-neural ectodermal cell types, such as the neural plate border (NPB). Using *Xenopus laevis*, we set out to determine whether SoxB1 transcription factors have a regulatory function in NPB formation. Here, we show that SoxB1 factors are necessary for NPB formation, and that prolonged SoxB1 factor activity blocks the transition from a NPB to a neural crest state. Using ChIP-seq, we demonstrate that Sox3 is enriched upstream of NPB genes in early NPB cells and in blastula stem cells. Depletion of SoxB1 factors in blastula stem cells results in downregulation of NPB genes. Finally, we identify Pou5f3 factors as potential Sox3 partners in regulating the formation of the NPB and show that their combined activity is needed for normal NPB gene expression. Together, these data identify a role for SoxB1 factors in the establishment and maintenance of the NPB, in part through partnership with Pou5f3 factors.

## Teaser

**Summary:** SoxB1 transcription factors are required for formation of the neural plate border in *Xenopus*, but then also serve as negative regulators of the transition from a neural plate border to neural crest state.

## INTRODUCTION

SoxB1 transcription factors (Sox1/2/3) are well known for their roles during early embryonic development in establishing and maintaining pluripotency and promoting neural fate specialization (Avilion et al., 2003; Kishi et al., 2000; Zhang and Cui, 2014). Prior to gastrulation, SoxB1 factors are expressed in cells of the embryo proper in vertebrate and invertebrate chordate species (Avilion et al., 2003; Buitrago-Delgado et al., 2018; Cattell et al., 2012; Okuda et al., 2006; Rex et al., 1997). Homozygous Sox2 murine mutants fail to develop past blastocyst stages, highlighting the importance of these transcription factors in cell survival during early embryonic development (Avilion et al., 2003). As gastrulation and lineage restriction commence, SoxB1 expression becomes restricted to the ectoderm and eventually to neuroectodermal cells at the onset of neurulation (Avilion et al., 2003; Buitrago-Delgado et al., 2018; Mizuseki et al., 1998; Okuda et al., 2006; Rex et al., 1997; Uy et al., 2012; Wood and Episkopou, 1999; York et al., 2023 preprint). During this period, the ectoderm is being patterned into three regions: neural plate, neural plate border and non-neural ectoderm (Sasai and De Robertis, 1997). Although SoxB1 factor expression ultimately becomes restricted to neural cells where they function redundantly to maintain a neural progenitor state (Bylund et al., 2003), SoxB1 factors are also expressed in non-neural ectodermal lineages. Lineage-tracing experiments in chick have shown that initially Sox2-positive cells contribute not only to the neural plate, but also the epidermis and neural plate border (Roellig et al., 2017), but whether SoxB1 factors play functional roles in establishing these non-neural ectodermal cell types remains unclear.

Neural plate border cells, which give rise to the neural crest, pre-placodal ectoderm, epidermis, and neural cell types, arise lateral to the neural plate during gastrulation (Groves and LaBonne, 2014; Pla and Monsoro-Burq, 2018; Streit, 2002; Thawani and Groves, 2020). BMP, Wnt and FGF signaling pathways have all been implicated in the formation of the neural plate border and promote the expression of neural plate border genes, including *pax3/7*, *zic1* and *msx1* (Garnett et al., 2012; Li et al., 2009; Luo et al., 2001; Marchal et al., 2009; Monsoro-Burq et al., 2003; Pla and Monsoro-Burq, 2018; Steventon et al., 2009; Tribulo et al., 2003; Yokota et al., 2017). These transcription factors, in turn, help to promote/stabilize expression of each other and to activate neural crest or placodal gene expression (de Croze et al., 2011; Hong and Saint-Jeannet, 2007; Li et al., 2009; Monsoro-Burq et al., 2005; Nichane et al., 2008; Sato et al., 2005). Although functional roles for neural plate border-specific transcription factors have been defined, less is known about transcriptional inputs outside of signaling cascades that contribute to the early establishment of this cell type.

SoxB1 factors are promising candidate transcription factors for regulating early neural plate border gene expression. Recent single-cell data have shown that cells expressing neural plate border genes do not form a unique cluster, but rather are present in neural and non-neural ectoderm clusters before neurulation (Williams et al., 2022), suggesting that these cells may express ‘canonical’ transcription factors for neural and non-neural ectodermal lineages. In alignment with this, recent genomic analysis has revealed Sox2 binding in neural plate border/early neural crest cells (Hovland et al., 2022; Roellig et al., 2017). These Sox2-enriched regions of chromatin close as cells transition from a neural plate border to a definitive neural crest state (Hovland et al., 2022). Additionally, many neural plate border factors, including *zic1* and *pax3/7*, also have functional roles in neural cell types, suggesting overlap in the transcriptional regulation of these cells (Aruga et al., 2002a,b; Lin et al., 2016). Given these findings, we hypothesized that SoxB1 factors may promote neural plate border gene expression during gastrulation.

Here, we investigate a role for SoxB1 factors in the formation of the neural plate border using *Xenopus laevis*. We find that SoxB1 factor expression overlaps with the forming neural plate border until neurulation and is lost in those cells as definitive neural crest cells form. Using morpholino-mediated depletion, we show that SoxB1 factors are necessary for neural plate border formation. Forced SoxB1 factor expression promotes and prolongs a neural plate border state, delaying the formation of neural crest cells. Through chromatin immunoprecipitation (ChIP) followed by sequencing (ChIP-seq) analyses, we find that Sox3 directly regulates expression of core neural plate border genes, and this transcriptional regulation begins in blastula stem cells. Finally, we provide evidence that Sox3 partners with Pou5f3 factors to promote neural plate border formation. Together, these results identify a key functional role for SoxB1 factors in the formation of the neural plate border.

## RESULTS

### Sox3 colocalizes with the neural plate border marker *pax3* through gastrulation

**Fig. 1.**
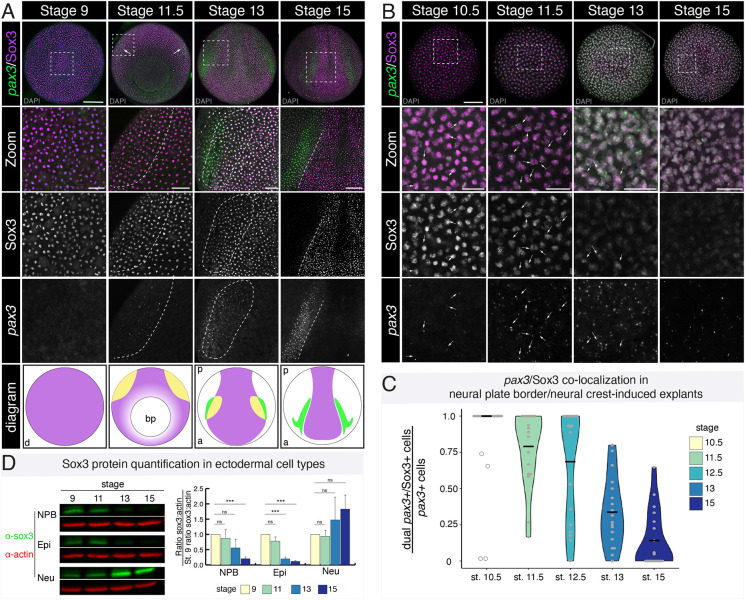
**Sox3 protein colocalization with *pax3* transcripts during gastrulation and colocalization decreases with the onset of neurulation.** (A) Whole embryos immunostained for Sox3 (magenta) and probed with HCR oligos for *pax3* (green) across multiple developmental stages (blastula through late neurula). DAPI is shown in gray in top two rows. Diagrams show domains of colocalization (yellow) and at each time point. Sox3 protein localization is displayed in magenta and *pax3* transcripts in green in diagrams. Dashed lines delineate regions of colocalization (stage 11.5, stage 13) or denote the boundary between Sox3-positive regions and *pax3*-positive region (stage 15). (B) Neural plate border/neural crest-induced explants immunostained for Sox3 (magenta) and probed with HCR oligos for intronic *pax3* (green), marking nascent transcripts, across multiple developmental stages (blastula through late neurula). DAPI is shown in gray in top two rows. Arrows indicate cells in which nascent *pax3* transcripts colocalize with nuclear Sox3 protein. Boxed areas are shown at higher magnification below. (C) Quantification of colocalization, as shown by violin plots, between Sox3 protein and nascent *pax3* transcripts over developmental time. Outlier data points are displayed as unfilled circles. Means are displayed as black horizonal bars. (D) Western blot for endogenous Sox3 protein (green) and actin (red) in neural plate border-induced explants, epidermal explants, and neural-induced explants at stages 9, 11, 13 and 15. Bar graph shows the quantification of western signal where the ratio of Sox3 to actin is normalized to stage 9. Error bars show s.d. ***P<0.001 (two-tailed *t*-test); a, anterior; bp, blastopore; d, dorsal; Epi, epidermis; p, posterior; Neu, neural; NPB, neural plate border; ns, not significant. Scale bars: 250 µm (A); 100 µm (A, zoom); 125 µm (B); 50 µm (B, zoom).

A role for SoxB1 factors in establishing the neural plate border would require they be expressed in that region during gastrulation. We therefore examined whether SoxB1 protein colocalizes with the key neural plate border factor *pax3*. As Sox2 and Sox3 are functionally redundant (Bylund et al., 2003) and have identical expression patterns at early stages of development (Fig. S1A), we focused on Sox3 localization, although expression levels of *sox2* at these stages are similar to that of *sox3* (Fig. S1B). A *Xenopus*-specific Sox3 antibody (Horr et al., 2023) was used in combination with a hybridization chain reaction (HCR) probe for *pax3* to mark the forming neural plate border (Choi et al., 2018). We set out to determine whether *pax3*-positive neural plate border cells were also Sox3 positive during gastrulation and neurulation. We observed that during gastrulation (stage 11.5) *pax3*-expressing cells are Sox3 positive (Fig. 1A). Co-expression of Sox3 and *pax3* appeared to decrease laterally by neural plate stages (stage 13) with little to no overlap by mid-neurula stages (stage 15) when definitive neural crest cells are present (Fig. 1A; Fig. S2E). Compared with expression levels of *sox2* and *sox3* from published data on dissected *Xenopus* neural plate border and neural crest cells (Plouhinec et al., 2017), we observed similar trends in expression levels of these genes with both factors being expressed in neural plate border cells (stage 12.5), but expression sharply decreasing in neural crest cells (stage 17; Fig. S1C).

To quantify more precisely the degree of cellular colocalization between Sox3 and *pax3*, we used a blastula stem cell explant system in which dissected blastula pole cells can be induced to form any cell type in the embryo given the appropriate developmental cues (Ariizumi and Asashima, 2001; Johnson et al., 2022). To induce a neural plate border state pharmacologically, we treated dissected explants with the small molecules CHIR99021 and K02288 (BMPi), a Wnt agonist and BMP antagonist, respectively (Huber and LaBonne, 2024). Neural plate border-induced explants were collected at time points corresponding with early gastrulation (stage 10.5) until mid-neurulation (stage 15) when they were immunostained for endogenous Sox3 and probed for nascent *pax3* transcripts using an intronic probe set (Fig. 1B; Fig. S2A). By examining nascent transcripts, we determined whether Sox3 protein is present in the nuclei of cells that are actively synthesizing *pax3* transcripts. Our colocalization analysis revealed that at early gastrula stages (stage 10.5) all nuclei with nascent *pax3* transcripts were Sox3 positive (Fig. 1C). We observed that *pax3* expression and nuclear Sox3 immunoreactivity remained highly correlated [79% colocalization (stage 11.5); 69% colocalization (stage 12.5)] until the start of neurulation (stage 13), when a decrease in colocalization (34%) was observed (Fig. 1C). By stage 15, only 14% of the *pax3*-positive cells were also Sox3 positive (Fig. 1C). Nascent *pax3* transcripts were infrequently observed in control epidermal explants (Fig. S2B-D). This analysis indicates that Sox3 protein is present in newly formed *pax3*-positive neural plate border cells during early to mid-gastrulation before decreasing at neurula stages as definitive neural crest genes become expressed (Fig. S2E).

We also examined changes in levels of Sox3 protein over time in three different ectodermal cell populations: neural plate border, epidermal cells and neural cells by western blot. Untreated blastula stem cell explants become epidermis as a result of endogenous BMP signaling (Wilson et al., 1997) and a neural state can be induced using a high dosage of BMPi (Johnson et al., 2022). Explants for each ectodermal cell type were collected at time points corresponding with blastula stage (stage 9) to mid-neurula (stage 15) and endogenous levels of Sox3 were assessed by western blot and quantified (normalized to actin) (Fig. 1D). Consistent with what we observe by immunofluorescence, the levels of Sox3 protein were highest in neural plate border cells during gastrulation (stage 11), with a significant decrease (80% decrease from stage 9; *P*=0.001) in Sox3 protein by mid-neurulation (stage 15). In contrast, Sox3 protein increased in neural-induced explants over time (Fig. 1D). Sox3 was also present at high levels in epidermal cells at stage 11, but decreased significantly (80% decrease from stage 9; *P*=0.001) by the start of neurulation (stage 13). Together with our immunofluorescence data, these findings demonstrate that at the onset of neural plate border formation nuclear Sox3 protein is present in these cells, but co-expression decreases during neurulation.

### SoxB1 transcription factors are necessary for neural plate border formation

**Fig. 2.**
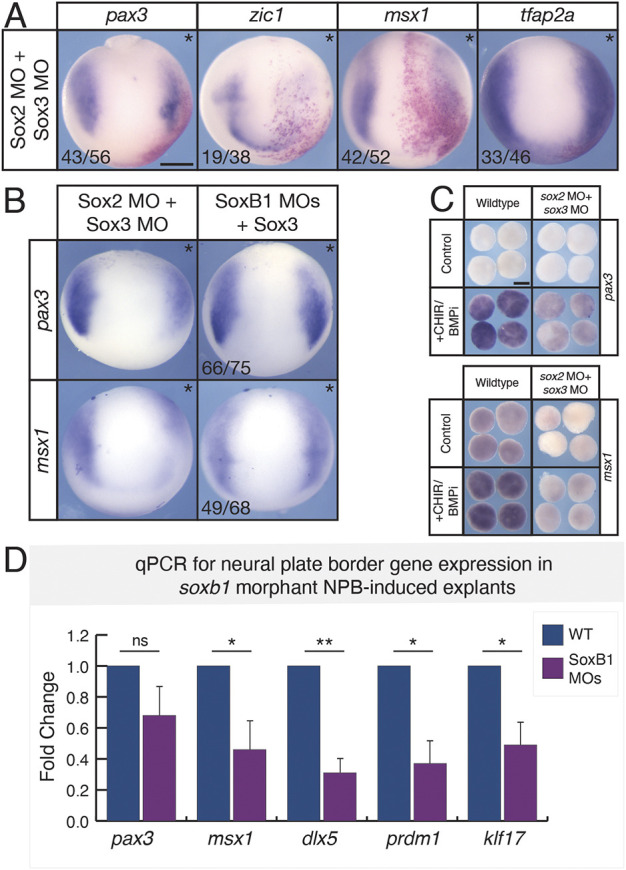
**SoxB1 factors are necessary for neural plate border formation.** (A) *In situ* hybridization for *pax3*, *zic1*, *msx1* and *tfap2a* in stage 12.5-13 embryos unilaterally injected with *sox2* and *sox3* morpholino (asterisks denote injected side). β-Galactosidase (red) was used as a lineage tracer. (B) *In situ* hybridization for *pax3* and *msx1* in stage 12.5-13 embryos unilaterally injected with *sox2* and *sox3* morpholinos or rescued embryos which were co-injected with *sox3* mRNA (asterisks denote injected side). Fluorescein dextran was used as a lineage tracer and embryos were pre-sorted for left/right side targeting. (C) *In situ* hybridization for *pax3* and *msx1* on wild-type and *soxb1* morphant neural plate border-induced explants (stage 12.5). (D) qPCR examining gene expression fold changes in neural plate border-induced explants (stage 12.5) comparing wild-type neural plate border-induced cells (blue) with *soxb1* morphant neural plate border-induced cells (magenta). Error bars show s.d. *P<0.05; **P<0.01; (two-tailed *t*-test). BMPi, BMP inhibitor (K02288); CHIR, CHIR99021 (Wnt agonist); MO, morpholino; NPB, neural plate border; ns, not significant. Scale bars: 250 µm.

The presence of Sox3 in forming neural plate border cells during gastrulation is consistent with a potential role for SoxB1 factors in regulating their formation. To determine whether SoxB1 factors are necessary for neural plate border gene expression, we designed and validated translation-blocking morpholinos targeting both alloalleles of *sox2* and *sox3* (Fig. S3). We next performed double morpholino-mediated depletion of Sox2 and Sox3 and examined neural plate border formation by *in situ* hybridization. We observed a loss of the neural plate border markers *pax3*, *zic1* and *msx1*, whereas expression of *tfap2a* remained unchanged (Fig. 2A; Fig. S4A). We found that neural plate border gene expression could be rescued by expressing a single SoxB1 factor (*sox3*) (Fig. 2B). We also examined the effects of SoxB1 depletion in blastula stem cells induced to a neural plate border state. We observed that the neural plate border genes *pax3* and *msx1* were poorly/weakly induced in SoxB1 morphants compared with controls (Fig. 2C; Fig. S4B). Using qPCR, we also found that expression of several additional neural plate border genes (*dlx5*, *prdm1*, *klf17*) was significantly reduced in double morphant neural plate border-induced explants (Fig. 2D) Together, these data indicate that SoxB1 factors are required for neural plate border formation.

### SoxB1 factor expression blocks the transition from a neural plate border to a neural crest state

**Fig. 3.**
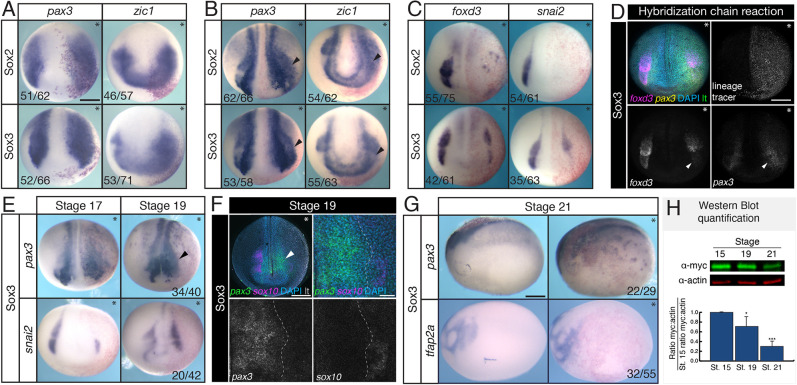
**SoxB1 expression blocks the transition from neural plate border to neural crest gene expression.** (A-C,E,G) *In situ* hybridization in embryos unilaterally expressing *sox2* or *sox3* mRNA (asterisks denote injected side). β-Galactosidase (red) was used as a lineage tracer. (A) *pax3* and *zic1* in early neurula embryos; (B) *pax3* and *zic1* in late neurula embryos (arrowheads denote expanded domain of expression); (C) *foxd3* and *snai2* in late neurula embryos; (E) *pax3* and *snai2* in stage 17 and stage 19 embryos unilaterally expressing *sox3* mRNA (arrowhead denotes expanded domain of expression); (G) *in situ* hybridization for *pax3* and *tfap2a* in stage 21 in embryos unilaterally expressing *sox3* mRNA. (D) Hybridization chain reaction for *foxd3* (magenta) and *pax3* (yellow) in embryos unilaterally expressing *sox3* mRNA (asterisks denote injected side). Myc antibody staining (green) was used as a lineage tracer. Arrowheads denote region with loss of *foxd3* expression and expanded *pax3* expression. (F) Hybridization chain reaction for *sox10* (magenta) and *pax3* (green) in embryos unilaterally expressing *sox3* mRNA (asterisk denotes injected side). Myc antibody staining (gray in top row) was used as a lineage tracer. Dashed lines delineate the boundary between *pax3*-positive cells and *sox10*-positive cells. (H) Time-course western blot with quantification for Myc-tagged Sox3 protein levels in the embryo where the ratio of Myc to actin is normalized to stage 15. *P<0.05; ***P<0.001 (two-tailed *t*-test). Error bars show s.d. lt, lineage tracer. Scale bars: 250 µm (A-G); 50 µm (F, zoom).

To determine whether SoxB1 factor activity promotes neural plate border gene expression, we performed gain-of-function experiments. mRNA encoding either Sox2 or Sox3 was injected into one blastomere at the two- or four-cell stage and embryos were assessed for changes in gene expression by *in situ* hybridization. Expression of either *sox2* or *sox3* resulted in expanded expression of *pax3* and *zic1* in early neurula embryos (stage 13; Fig. 3A). We next wanted to determine whether prolonged SoxB1 activity extended neural plate border gene expression in the embryo. Our western blot and imaging analyses indicated that Sox3 protein is largely absent in neural crest cells by the middle of neurulation (stage 15), so we examined neural plate border gene expression at this stage and later. We found that expression of both *pax3* and *zic1* was expanded into the presumptive neural crest domain in mid-neurula embryos (Fig. 3B, arrowheads). We next examined neural crest gene expression at this stage and observed a loss of neural crest, as evidenced by loss of *foxd3* and *snai2* gene expression (Fig. 3C; Fig. S5A) (Buitrago-Delgado et al., 2018; Rogers et al., 2009; Wakamatsu et al., 2004). Supporting these results, when we examined expression of *foxd3* and *pax3* in *sox3*-expressing embryos using HCR, we found that *pax3*-positive cells were present in the domain where *foxd3* expression had been lost (Fig. 3D, arrowheads). We also examined markers for other ectodermal lineages (epidermis, placode and neural) in SoxB1-expressing embryos, and found that expression of those lineage markers was also decreased (Fig. S5B) (Rogers et al., 2009). These data suggest that SoxB1 activity causes cells that would normally become definitive neural crest to instead be retained in a neural plate border state.

If prolonged SoxB1 activity prevents the transition from a neural plate border to a neural crest state, it is possible that the neural crest may recover later in development once SoxB1 proteins turn over, similar to what we observe during neurulation in wild-type embryos (Fig. 1). To test this, we performed *in situ* hybridization for *pax3* and *snai2* on *sox3*-injected embryos collected at stage 19. We found that *pax3* expression continues to be laterally expanded at this stage, whereas *snai2* expression either remains lost (35%) or has partially recovered (48%), but displays delayed migration (Fig. 3E; Fig. S5C). We also examined localization of *pax3-* and *sox10*-expressing cells in *sox3*-injected embryos (stage 19) using HCR. We observed that sox*10* expression is absent in the domain of expanded *pax3* expression (Fig. 3F). By stage 21, wild-type neural crest cells have delaminated and begun to migrate (Theveneau and Mayor, 2012). When we examined expression of *pax3* and *tfap2a*, a marker for migrating neural crest cells, we found that anterior *pax3* expression is medially restricted and *tfap2a* expressing cells are present, also indicating a recovery of the neural crest (Fig. 3G; Fig. S5C). Western blot analysis indicated that Myc-tagged Sox3 protein levels were reduced by 30% (*P*=0.04) at stage 19 and 70% (*P*=1.2×10^−4^) at stage 21 relative to levels at stage 15 (Fig. 3H). These data indicate that transitioning from a neural plate border to neural crest state is correlated with decreased levels of SoxB1 protein.

### Sox3 is enriched upstream of neural plate border genes

**Fig. 4.**
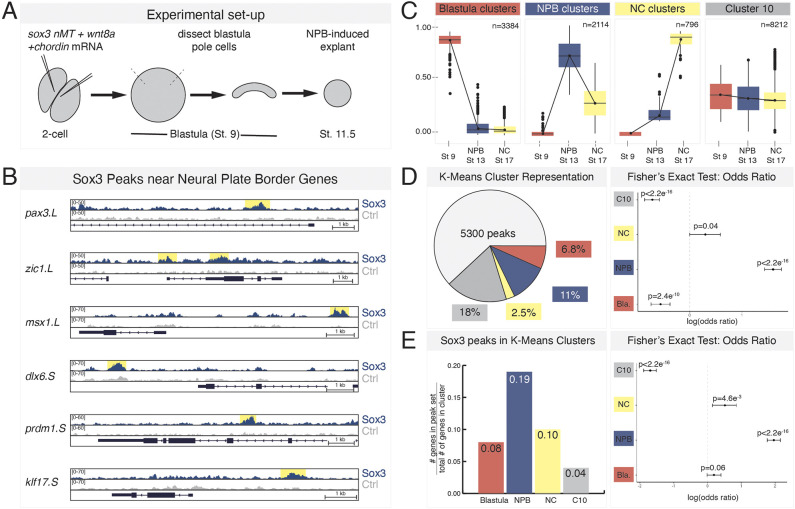
**ChIP-seq in early neural plate border cells reveals Sox3 enrichment at multiple neural plate border genes.** (A) Schematic of experimental design for Sox3 ChIP-seq experiments in neural plate border-induced explants. (B) Genome browser view of Sox3 peaks at loci for neural plate border genes (*pax3*, *zic1*, *msx1*, *dlx6*, *prdm1* and *klf17*). (C) Representative k-means clusters for transcriptome data for blastula stem cells (stage 9), neural plate border-induced explants (stage 13), and neural crest-induced explants (stage 17) (adapted from York et al., 2023 preprint). (D) Percentage of Sox3 peaks (5300 peaks total) associated with k-means cluster genes and accompanying forest plot for odds ratio. (E) Percentage of genes in k-means clusters with associated Sox3 ChIP peaks and accompanying forest plot for odds ratio. Red, blastula cluster; blue, neural plate border (NPB) cluster; yellow, neural crest (NC) cluster; gray, cluster 10 (C10). Ctrl, control; nMT, N-terminal Myc-tag.

To determine whether Sox3 directly regulates neural plate border gene expression, we performed ChIP-seq in mid-gastrula (stage 11.5) explants expressing Myc-tagged *sox3*, at near endogenous protein levels (Fig. S6A), that had been induced to a neural plate border state with *wnt8a* and *chordin* mRNA (LaBonne and Bronner-Fraser, 1998) (Fig. 4A). We validated that these *sox3*-injected explants were bona fide neural plate border cells by assaying for *pax3* expression (Fig. S6B). Peak calling identified 5300 peaks enriched for Sox3 binding. When we examined genomic regions of known neural plate border genes (*pax3*, *zic1*, *msx1*, *dlx6*, *klf17*, *prdm1*), we observed Sox3 enrichment upstream (∼5 kb or less), or in putative regulatory regions of introns of these genes (Fig. 4B).

We next wanted to determine whether Sox3 enrichment occurs more frequently at neural plate border genes then at non-neural plate border genes. To do so, we utilized previously published k-means analysis of transcriptome data from *Xenopus* blastula stem cells (stage 9) and neural plate border/neural crest-induced explants (stage 13/17) (Godichon-Baggioni et al., 2019; Rau and Maugis-Rabusseau, 2018; York et al., 2024). Specifically, we utilized clusters that display distinct signatures for blastula (*pou5f3.3*, *foxi2*), neural plate border (*pax3*, *zic1*) and neural crest (*foxd3*, *snai2*) genes, and a fourth cluster, cluster 10 (C10), consisting of genes lacking dynamic expression changes, that includes many housekeeping genes (*cox1*, *gapdh* and *ubc*; Silver et al., 2008) (Fig. 4C). We used these gene lists to assess whether Sox3 occupancy was more highly enriched in neural plate border genes (blue) than non-neural plate border genes (red, yellow and gray).

We compared the list of genes associated with our Sox3 ChIP peaks, as defined by the nearest transcription start site, to these different k-means clusters and found that, of the 5300 peaks, 11% (*n*=596) were associated with genes in the neural plate border cluster (Fig. 4D). We calculated an odds ratio for this value using a Fisher's exact test and found this association to be highly significant (odds ratio=3.02; *P*<2.2×10^−16^). In contrast, the other three clusters were not significantly enriched for Sox3, as determined by Fisher's exact testing (Fig. 4D). We also performed the reciprocal analysis and investigated how many genes in each cluster group had an associated Sox3 ChIP peak. We found that, of the 2114 genes in the neural plate border cluster, 19% had a corresponding Sox3 peak (*n*=400). In contrast, Sox3 peaks only represented 10% (NC), 8% (blastula) and 4% (C10) of the genes in the other three clusters (Fig. 4E). We again performed Fisher's exact tests on these data and observed a high odds ratio (odds ratio=3.39; *P*<2.2×10^−16^) for the neural plate border cluster, suggesting that Sox3 is more highly enriched at neural plate border genes than at non-neural plate border genes.

Finally, we performed DESeq2 on previously published transcriptome data from stage 13 epidermal versus neural plate border-induced explants (York et al., 2023 preprint) and identified genes with differential expression (*P*_adj_<0.05, log FC>1.5) in both cell types (Fig. S7). Of the 1675 differentially expressed genes in neural plate border cells, 452 were found to have an associated Sox3 ChIP peak (odds ratio=3.88; *P*<2.2×10^−16^) (Fig. S7). In contrast, Sox3 was not significantly enriched at differentially expressed epidermal genes (odds ratio=0.74; *P*=4.2×10^−5^). Overall, this ChIP-seq data analysis indicates that Sox3 is highly enriched at regulatory regions for neural plate border genes.

### Sox3 regulates neural plate border gene expression in blastula stem cells

**Fig. 5.**
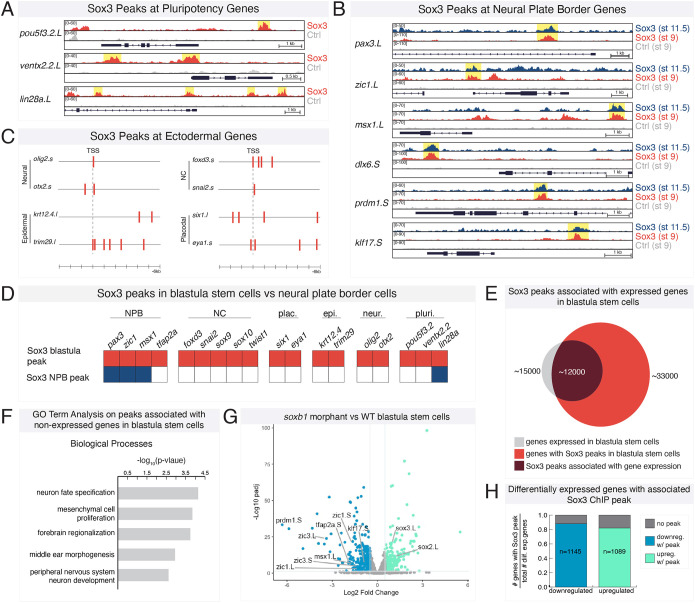
**Sox3 is enriched upstream of neural plate border and other ectodermal genes in blastula stem cells.** (A) Genome browser view of Sox3 ChIP peaks at loci for pluripotency genes (*pou5f3.2*, *ventx2.2* and *lin28a*). (B) Genome browser view of Sox3 ChIP peaks at loci for neural plate border genes (*pax3*, *zic1*, *msx1*, *dlx6*, *prdm1* and *klf17*) in blastula stem cells (red) and neural plate border cells (blue). (C) Diagram of Sox3 peaks (red bars) near the transcription start site (TSS) of neural (*olig2*, *otx2*), epidermal (*krt12.4*, *trim29*), neural crest (NC; *foxd3*, *snai2*) and placodal genes (*six1*, *eya1*) in blastula stem cells. (D) Graphical summary of genome occupancy in blastula stem cells and neural plate border cells with colored boxes (red/blue) indicating Sox3 enrichment. (E) Venn diagram showing overlap (maroon) between blastula stem cell transcriptome (gray) and Sox3 peaks in blastula stem cells (red). (F) GO term analysis for annotated genes associated with Sox3 peaks that are not expressed in blastula stem cells. (G) Volcano plot for differentially expressed genes between wild-type blastula stem cells and *soxb1* morphant blastula stem cells. Genes downregulated in *soxb1* morphants are shown in blue and include several neural plate border genes. Upregulated genes are shown in green. (H) Percentage of differentially expressed genes with an associated blastula stage Sox3 peak. epi., epidermis; NC, neural crest; neur., neural; NPB, neural plate border; plac., placode; pluri., pluripotency.

Previous work from our lab has shown that several neural plate border genes are initially expressed in blastula stem cells (Buitrago-Delgado et al., 2015; York et al., 2023 preprint). Given that SoxB1 factors are also robustly expressed in blastula stem cells, we next investigated whether SoxB1 factors may be regulating expression of neural plate border genes at blastula stages. We performed ChIP-seq in blastula stem cells expressing Myc-tagged *sox3* mRNA at near-endogenous protein levels (Fig. S8). Peak calling identified ∼124,000 Sox3 peaks. As expected, we found Sox3 enriched upstream of many pluripotency genes, including *pou5f3* factors (Oct3/4), *ventx2.2* (functional equivalent of Nanog) and *lin28a* (Fig. 5A). Sox3 enrichment was also observed near neural plate border genes, and we found that these peaks were highly correlated with the Sox3 peaks identified in our neural plate border ChIP data set, occupying the same genomic loci (Fig. 5B). Interestingly, we also observed Sox3 enrichment at blastula stages near genes for other ectodermal cell types: neural, epidermal, placodal, and neural crest (Fig. 5C); however, Sox3 occupancy was not maintained for these genes in neural plate border cells (Fig. 5D). This temporal loss of Sox3 occupancy was also observed for *tfap2a*, which may explain why *soxb1* morphant embryos still express *tfap2a* (Fig. 2A). These data suggest that SoxB1 factors, which are known to function as pioneer factors (Soufi et al., 2015), bind to genes associated with many ectodermal lineages in blastula stem cells, but by mid-gastrula stages Sox3 occupancy is predominantly retained at neural plate border genes.

Additionally, we noted that some Sox3 peaks (stage 9) were associated with genes that are not expressed at blastula stages (e.g. *pax3*, *olig2*, *six1*), consistent with a role for SoxB1 factors in poising genes for later expression (Fig. S9). Of the ∼33,000 Sox3 peaks associated with annotated genes, only 36% of these genes are expressed at blastula stage (Fig. 5E). Gene ontology (GO) analysis of the 64% of genes not expressed in blastula stem cells showed enrichment for GO terms associated with central and peripheral nervous system development (Fig. 5F), cell types in which SoxB1 factors play key roles in later in development or postnatally (Bani-Yaghoub et al., 2006; Carmona-Fontaine et al., 2008; Kautzman et al., 2018; Kuhbandner, 2018; Parrinello et al., 2010; Roberts et al., 2017; Stevanovic et al., 2021; Torres-Mejia et al., 2020; Wang et al., 2022). Together, these analyses provide evidence that SoxB1 factors may poise genes involved in the development of the neural plate border and other ectodermal lineages as early as blastula stages.

As our results demonstrated that Sox3 directly binds neural plate border genes in blastula stem cells, we next examined whether SoxB1 factors regulate expression of these genes at this stage. We performed RNA sequencing (RNA-seq) on dissected blastula stem cells (stage 9) depleted for *sox2* and *sox3* through use of morpholinos. DESeq2 was used to identify genes differentially expressed between *soxb1* morphant and control blastula stem cells. We found 2235 genes to be differentially expressed (*P*_adj_<0.05) with approximately equal numbers up- and downregulated (Fig. 5G,H). Approximately 85% of differentially expressed genes had an associated blastula stage Sox3 ChIP peak (Fig. 5H). Among the genes downregulated in *soxb1* morphant cells were several canonical neural plate border factors, including *zic1*, *msx1* and *tfap2a* (Fig. 5G). We also observed an upregulation of *sox2* and *sox3* gene expression in *soxb1* morphants cells. These data suggest that, not only do SoxB1 factors bind to regulatory regions of neural plate border genes, but they also promote expression of a subset of these genes at blastula stages.

### Pou5f3 factors are co-expressed with SoxB1 factors in neural plate border cells

**Fig. 6.**
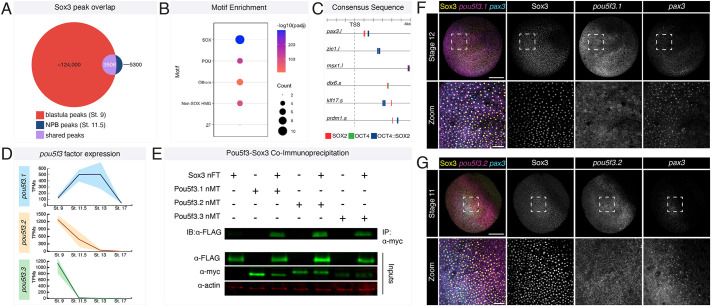
**Motif analysis identifies POU factors as potential SoxB1 transcriptional partners involved in neural plate border formation.** (A) Venn diagram showing overlap (purple) between Sox3 blastula stage peaks (red) and Sox3 neural plate border (NPB) cell peaks (blue). (B) Dot plot showing motif enrichment for the shared category (purple) of Sox3 peaks. Motifs for POU transcription factors are highly enriched. (C) Localization of Sox2, Oct4 and Oct4-Sox2 motifs upstream of neural plate border gene transcription start sites (TSS). (D) TPM plots for *pou5f3* factor expression from blastula stem cells (stage 9), neural plate border-induced explants (stage 11.5, stage 13) and neural crest-induced explants (stage 17). (E) Co-immunoprecipitation of Sox3 with Pou5f3.1, Pou5f3.2 and Pou5f3.3 factors. (F) Wild-type embryo (stage 12) immunostained for Sox3 (yellow) and probed with HCR oligos for *pou5f3.1* (magenta) and *pax3* (cyan). (G) Wild-type embryo (stage 11) immunostained for Sox3 (yellow) and probed with HCR oligos for *pou5f3.2* (magenta) and *pax3* (cyan). Boxed areas in F,G are shown at higher magnification below. IB, immunoblot; IP, immunoprecipitated; nFT, N-terminal FLAG tag; nMT, N-terminal Myc tag; NPB, neural plate border; ZF, zinc finger. Scale bars: 250 µm (F,G); 100 µm (F,G, zoom).

SoxB1 factors require a transcriptional partner to regulate gene expression (Kondoh and Kamachi, 2010). To identify candidate SoxB1 transcriptional partners involved in initiating and maintaining neural plate border gene expression, we identified Sox3 peaks that were shared between our Sox3 blastula stage (stage 9) and neural plate border (stage 11.5) ChIP-seq datasets. We found ∼3500 shared peaks, representing 66% of the total peaks in the neural plate border dataset (Fig. 6A). Motif analysis of these shared peaks identified high enrichment for Sox factor, non-Sox HMG box factor (TCF/LEF), and POU factor motifs. As we noted that an Oct4-Sox2 consensus sequence was present in approximately 25% of shared peaks (Fig. 6B; Fig. S10A), we examined whether the Sox3 peaks associated with common neural plate border genes possessed Oct4-Sox2 consensus sequences. We found that most of these genes had at least one Oct4-Sox2 consensus site (Fig. 6C). We also performed motif analysis on shared regions of Sox3 enrichment for the genes within the four k-means clusters (Fig. 4C) and found that POU motifs, including motifs for Oct4-Sox2, were enriched in the neural plate border cluster (Fig. S10B). These findings are consistent with recent assay for transposase-accessible chromatin with sequencing (ATAC-seq) data, which found enrichment for Oct4-Sox2 motifs in avian neural plate border/early neural crest cells (Hovland et al., 2022).

Given that combined activity of Pou5f (Oct3/4) and SoxB1 factors is important for maintenance of pluripotency in mouse and human embryonic stem cells (Avilion et al., 2003; Boyer et al., 2005; Dailey and Basilico, 2001; Loh et al., 2006; Matin et al., 2004; Nichols et al., 1998; Niwa et al., 2000; Rodda et al., 2005; Wang et al., 2007), we wished to determine whether they jointly regulate neural plate border formation. *Xenopus laevis* has three Pou5f3 homologs, *pou5f3.1* (*pou5f3*), *pou5f3.2* and *pou5f3.3*. *Pou5f3.3* is highly expressed in blastula stem cells, with low levels of expression in neural plate border cells, whereas *pou5f3.1* and *pou5f3.2* are expressed in both blastula stem cells and neural plate border cells (Fig. 6D; Fig. S11A) (Morrison and Brickman, 2006; Plouhinec et al., 2017; York et al., 2023 preprint). Through co-immunoprecipitation experiments, we found that all three Pou5f3 factors can physically interact with Sox3 (Fig. 6E). We next assessed whether *pou5f3.1/2* and Sox3 are co-expressed in forming neural plate border cells using HCR probes for *pou5f3.1*/*pou5f3.2* and *pax3* and immunostaining for Sox3. Although Sox3 and *pou5f3.1/2* are broadly expressed in gastrulating embryos, we observed co-expression of these factors within *pax3*-expressing neural plate border cells (Fig. 6F,G). Consistent with this, when we used HCR probes for *pou5f3.1*/*pou5f3.2* and immunostaining for Sox3 in neural plate border-induced explants, we observed co-expression these factors (Fig. S11B). These data indicate that SoxB1 factors and Pou5f3 factors are co-expressed in early neural plate border cells and could be functioning together to regulate formation of these cells.

### Combined activity of Sox3 and Pou5f3 is required for neural plate border formation

**Fig. 7.**
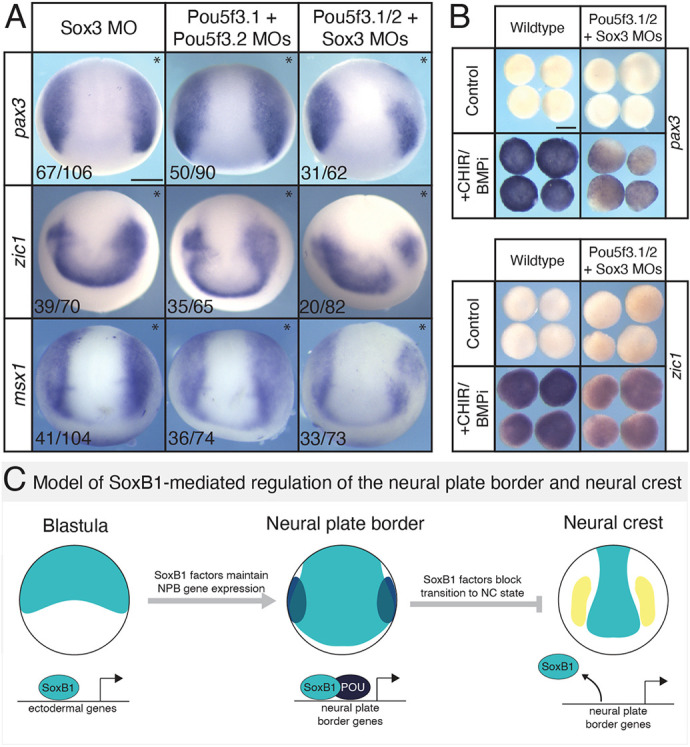
**Combined activity of Pou5f3 and SoxB1 transcription factors is required for neural plate border formation.** (A) *In situ* hybridization for *pax3*, *zic1* and *msx1* in early neurula embryos injected unilaterally (asterisks mark the injected side) with Sox3 morpholino, Pou5f3.1+Pou5f3.2 morpholinos, or Sox3+Pou5f3.1+ Pou5f3.2 morpholinos. Fluorescein dextran was used as a lineage tracer and embryos were presorted for left/right side targeting. (B) *In situ* hybridization for *pax3* and *zic1* on wild-type and triple-morphant neural plate border-induced explants (stage 13). (C) Proposed model for SoxB1-mediated regulation of the neural plate border and neural crest. SoxB1 factors (teal) bind near ectodermal genes in blastula stem cells and promote or poise gene expression. SoxB1 factors, in partnership with Pou5f transcription factors (navy), maintain neural plate border gene (blue) expression throughout gastrulation. Downregulation of SoxB1 in neural plate border cells is required for neural crest (yellow) gene expression. MO, morpholino; NC, neural crest; NPB, neural plate border. Scale bars: 250 µm (A); 125 µm (B).

To determine whether the combined activity of SoxB1 and Pou5f3 is required for neural plate border formation, we inhibited expression of *sox3*, *pou5f3.1* and *pou5f3.2* using morpholino-mediated depletion and examined the expression of neural plate border genes at the end of gastrulation. Depletion of *sox3* alone resulted in modest expansion of neural plate border gene expression (*pax3*, *zic1*, *msx1*) (Fig. 7A), but was also accompanied by upregulation of *sox2* and *sox3* (Fig. S12), as also observed in our blastula RNA-seq data (Fig. 5G). This may suggest that Sox2 is overcompensating for the downregulation of Sox3, resulting in a net gain of SoxB1 activity and the observed phenotypes. Combined loss of *pou5f3.1* and *pou5f3.2* also resulted in a small expansion of neural plate border gene expression in more than half of embryos. Despite these two depletions resulting in expanded neural plate border expression in most cases, triple morphants (Sox3+ Pou5f3.1*+*Pou5f3.2 morpholinos) were found to frequently display a partial loss of neural plate border gene expression (Fig. 7A). We noted that *pax3* and *msx1* appeared to be more sensitive to loss of these transcription factors than was *zic1*, which only displayed a partial loss 24% of the time (Fig. S13). In further support of these findings, when we pharmacologically induced blastula stem cell explants to a neural plate border state, we found that triple morphant explants were poorly/weakly induced compared with controls (Fig. 7B; Fig. S13), suggesting that the combined activity of Sox3 and Pou5f3 transcription factors is essential for maintenance of neural plate border gene expression.

## DISCUSSION

We set out to determine whether SoxB1 transcription factors play a role in establishing the neural plate border. Our ChIP-seq data provides evidence that SoxB1 factors function to poise a broad range of ectodermal genes in blastula stem cells, including neural plate border genes. Through knockdown experiments, we determined that SoxB1 factors regulate the expression of many neural plate border genes at blastula stages and are required for neural plate border formation. We also show that SoxB1 factors promote neural plate border gene expression and that downregulation of SoxB1 factors following neural plate border establishment is necessary for the transition from a neural plate border to a neural crest state. Our ChIP-seq data provide evidence that regulation of neural plate border gene expression by Sox3 is likely direct. Finally, we demonstrate that combined activity of Sox3 and Pou5f3 transcription factors is necessary for maintaining proper neural plate border gene expression (Fig. 7C).

### Transitioning from a neural plate border state to a neural crest state

Embryonic development relies upon precise regulation of developmental transitions to establish new cell types. Here, we identify SoxB1 factors as negative regulators of the transition from a neural plate border to neural crest state. Prior work suggests that there is a delicate balance between levels of neural plate border transcription factors and promotion of neural crest gene expression. In *Xenopus*, gain-of-function experiments for *pax3* and *zic1* have shown that whereas low levels of expression promote neural crest formation, high levels inhibit neural crest formation and promote hatching gland formation (Hong and Saint-Jeannet, 2007). Here, we show that expression of neural plate border genes is expanded and prolonged in *soxb1*-injected embryos and neural crest formation is delayed. These results are similar to *pax3*/*zic1* gain-of-function experiments (high levels) and further emphasize the importance of strict temporal regulation of these neural plate border factors as well as maintenance of specific threshold protein levels.

Understanding how SoxB1 factors are regulated at the neural plate border is important for understanding how this state transition occurs. It is likely that the precise spatiotemporal control of *soxb1* expression levels and expression domain is dependent upon feedback loops from neural plate border and neural crest genes. For example, *sox2* expression in the neural plate has been found to expand in *pax3*, *zic1*, *msx1* and *tfap2a* morphant embryos (Hong and Saint-Jeannet, 2007; Luo et al., 2003; Monsoro-Burq et al., 2005). Similarly, knockdown or use of a dominant negative form of the neural crest genes *myc*, *snai2*, *sox9* or *foxd3* results in an expansion of *sox2* expression (Bellmeyer et al., 2003; LaBonne and Bronner-Fraser, 2000; Langer et al., 2008; Sasai et al., 2001; Spokony et al., 2002). Interestingly, knockdown of Prdm1 in chick leads to a loss of *Sox2* but an expansion of *Sox3* (Prajapati et al., 2019), although it is unclear why these two SoxB1 factors respond differently to loss of Prdm1. Overall, these findings, along with our data, support a model in which SoxB1 factors promote expression of neural plate border-linked genes in blastula stem cells, but as the expression levels of those factors increase during neural plate border formation, they restrict *soxb1* expression medially to the neural plate. The restriction of SoxB1 factors to the neural plate is further reinforced by the expression of neural crest factors.

### SoxB1 factors and poising ectodermal genes in stem cells

SoxB1 transcription factors are well known for their roles in establishing a pluripotent state in cell culture (Takahashi and Yamanaka, 2006); however, less is known about their specific functions in pluripotent cells *in vivo*. Recent work has shown that there are several differences in Sox2 target gene occupancy and gene regulation between naive embryonic stem cells and e4.5 epiblast cells (Li et al., 2023), emphasizing a need to conduct *in vivo* studies. Akin to what has been observed in mouse epiblast cells, we also observe SoxB1 factor binding to pluripotency genes and ectodermal-specific genes in *Xenopus* blastula stem cells. Furthermore, both data sets show that binding to pluripotency genes is largely lost, but ectodermal gene occupancy (in our case neural plate border gene occupancy, specifically) is retained (Li et al., 2023). Each dataset suggests that ectodermal genes are poised by SoxB1 transcription factors in blastula stem cells/epiblast cells, but Li et al. have also hypothesized that Sox2 may be capable of ‘pilot binding’ whereby it binds to a regulatory element, but does not open chromatin until receiving differentiation cues. Further ATAC-seq studies in *Xenopus* are needed to assess whether ‘pilot binding’ is occurring at these Sox3-poised genes as well and to determine which cues are responsible for the shift from pilot to pioneer activity.

### SoxB1-Pou5f3 regulation of neural plate border and neural crest formation

Sox and POU transcription factors directly interact to regulate many aspects of embryonic development. In neural progenitor cells, Sox2 and the class III POU factor Brn2 (Pou3f2) synergize to activate nestin expression (Tanaka et al., 2004). Additionally, Sox2 and Oct4 (Pou V factor; Pou5f1) are well known for regulating pluripotency in mammalian embryonic stem cells (Avilion et al., 2003; Boyer et al., 2005; Lodato et al., 2013). We identify another role for SoxB1 and Pou5f3 factors in regulation of neural plate border gene expression. Recent work in chick has identified a role for the SOX2-OCT4 heterodimer in establishing a neural crest epigenomic signature and showed that prolonged heterodimer expression leads to maintenance of *PAX7* expression in what appear to be migrating neural crest cells (Hovland et al., 2022). Although these data may seem to conflict with our findings, it is possible that prolonged *PAX7* expression is reflective of maintaining a neural plate border state rather than an early neural crest state. *PAX7* is one of the earliest markers for this cell population in chick (Prasad et al., 2020). Analysis of additional markers is necessary to distinguish between these two possibilities. Additionally, work in human cell culture has shown a global reduction in Sox2 binding in neural plate border cells relative to blastula cells (Hovland et al., 2022), which we also observe in *Xenopus*. However, we observed that Sox3 binds to neural plate border and neural crest genes in blastula stem cells, whereas they observed SOX2-OCT4 binding to neural crest *cis*-regulatory regions upon neural crest specification *in vitro* (Hovland et al., 2022). ATAC-seq in mouse has shown that the *cis*-regulatory landscape of Oct4^+^ neural crest progenitor cells and epiblast stem cells are highly correlated, similar to our findings in *Xenopus* (Zalc et al., 2021). It is possible that noted differences in chromatin accessibility across these datasets is due to species-specific differences or a difference in timing of genome occupancy experiments. Still, a major unanswered question in all of these systems is what mediates changes in transcription factor occupancy as cells transition from one cell state to another.

It is also worth noting that SoxB1 factors and Pou5f3 factors have different effects on neural crest gene expression. The neural crest is expanded in *pou5f3*-injected *Xenopus* embryos (York et al., 2023 preprint), suggesting that Pou5f3 factors may have functions outside a SoxB1-Pou5f3 dimer during neural plate border/neural crest development. An interesting hypothesis is that Pou5f3 factors may be interacting with SoxE proteins in neural crest cells to promote neural crest gene expression. It is known that SoxB1 transcription factors interact with Pou5f3 factors through the highly conserved HMG domain (Remenyi et al., 2003), making it possible that Pou5f3 factors could also directly interact with SoxE factors. Recently it has been shown that replacing alanine 61 in the HMG domain of Sox2 with a valine increases Oct4-Sox2 heterodimer stability (MacCarthy et al., 2023). Interestingly, Sox8 and Sox9, SoxE factors, have a valine at residue 61 in their HMG domain, suggesting that SoxE may form a more stable complex with Pou5f3 factors than SoxB1 factors and together promote neural crest formation. A possible model would be that Pou5f3 factors cooperate with SoxB1 factors to promote neural plate border formation, but a shift in binding cooperativity and function occurs as SoxE factors are expressed, an area of current investigation. This model would also provide a possible explanation for why genome occupancy of Sox3 is lost at neural crest genes in neural plate border cells, as it is possible this chromatin remains open throughout gastrulation and occupancy of Sox3 is gradually switched to SoxE factors at these sites.

### Candidate SoxB1 transcriptional partners important for neural plate border establishment

It is also likely that SoxB1 factors are also partnering with other transcription factors, in addition to Pou5f3 factors, to promote or refine neural plate border gene expression. Our motif analysis also found TCF/LEF motifs to be highly enriched at Sox3 peaks. SoxB1 factors can directly bind to β-catenin and other Sox factors have been shown to bind TCF/LEF transcription factors (Chen et al., 2008; Sinner et al., 2004; Zorn et al., 1999). In some cellular contexts, Sox proteins function to promote or enhance Wnt signaling through stabilization of β-catenin or by synergizing with β-catenin to promote Wnt target gene expression (Chen et al., 2008; Sinner et al., 2004). However, in other cellular contexts, Sox proteins repress canonical Wnt signaling. It has been hypothesized this is due to competition between Sox proteins and β-catenin for TCF/LEF binding, preventing target gene expression (Akiyama et al., 2004). It has also been shown that Sox proteins can promote β-catenin degradation (Akiyama et al., 2004; Topol et al., 2009). Given that Wnt signaling is essential for the establishment of the neural plate border, it is possible that SoxB1 factors may additionally function to regulate Wnt signaling, positively or negatively, in neural plate border cells.

### SoxB1 factors and the evolution of the neural crest

SoxB genes are present in the genome of basal metazoans and are hypothesized to have arisen in the last common ancestor of metazoans (Larroux et al., 2006). Both invertebrate chordates and vertebrates possess SoxB1 transcription factors (Heenan et al., 2016). The invertebrate chordate amphioxus possesses lateral neural border cells, but lacks neural crest. These cells are hypothesized to be the homolog to the vertebrate neural plate border and give rise to sensory neurons. In amphioxus embryos, *SoxB1* factors are expressed in the neural plate where expression persists in the neural tube until larval stages (Meulemans and Bronner-Fraser, 2007). Interestingly, SoxB1-a expression overlaps with lateral neural border gene expression in this species. Likewise, in lamprey, a jawless vertebrate, SoxB1a expression overlaps with neural plate border before restricting to the neural plate (Uy et al., 2012; York et al., 2023 preprint). SoxB1-expressing cells are notably absent from the neural crest in lamprey, as in *Xenopus*, and are restricted to the neural tube (Uy et al., 2012). These expression data indicate that SoxB1 factors are expressed in the neural plate border in both invertebrate chordates and vertebrates, suggesting that SoxB1-dependent regulation of neural plate border gene expression may be evolutionarily conserved. As amphioxus lack neural crest, it would be interesting to know SoxB1 whether expression persists in lateral neural border cells. This could suggest that changes in SoxB1 *cis*-regulatory elements were needed for the downregulation of *SoxB1* expression in these cells for the neural crest to form. It is possible that modulation of SoxB1 expression in those cells may be one important distinguishing feature of the vertebrate versus nonvertebrate chordate border.

## MATERIALS AND METHODS

### Animals

All animal procedures were approved by the Institutional Animal Care and Use Committee, Northwestern University, and are in accordance with the National Institutes of Health's Guide for the Care and Use of Laboratory Animals.

### Embryological methods

Wild-type *Xenopus laevis* embryos were obtained using standard methods and cultured in 0.1× Marc's Modified Ringer's Solution (MMR) [0.1 M NaCl, 2 mM KCl, 1 mM MgSO_4_, 2 mM CaCl_2_, 5 mM HEPES (pH 7.8), 0.1 mM EDTA] until the desired stages (Nieuwkoop and Faber, 1994). Embryos or blastula stem cell explants (also known as animal pole cell explants) used for *in situ* hybridization, HCR or immunofluorescence were fixed in 1× MEM [100 mM MOPS (pH 7.4), 2 mM EDTA, 1 mM MgSO_4_] with 3.7% formaldehyde and dehydrated in methanol prior to use. Embryos or blastula stem cell explants that underwent *in situ* hybridization were processed as described by LaBonne and Bronner-Fraser (1998) and imaged using an Infinity 8-8 camera (Teledyne Lumenera). Results are representative of a minimum of three biological replicates.

Microinjection of mRNA or morpholinos was carried out at the 2- to 8-cell stage. mRNA was synthesized using an mMessage mMachine SP6 Transcription Kit (Invitrogen) and translation efficiency assessed by western blot. Either β-galactosidase mRNA or fluorescein dextran were co-injected as a lineage tracer. Approximately 10-25 ng of translation-blocking morpholinos (Gene Tools) were injected per cell. Morpholino sequences were as follows: *sox2*: 5′-TCTCCATCATGCTGTACAT-3′; *sox3*: 5′-TCCAACATGCTATACATTTGGAG-3′; *pou5f3.1.L*: 5′-CCTATACAGCTCTTGCTCAAATC-3′; *pou5f3.1.S*: 5′- GATTAAACATGATCTGTTGTCCG-3′; *pou5f3.2.L*: 5′- CCAAGAGCTTGCAGTCAGATC-3′; *pou5f3.2.S*: 5′- GCTGAACCCTAGAATGACCAG-3′.

### Blastula stem cell explant assays

Animal pole cells were manually dissected using forceps at blastula stage (stage 9). Manipulated embryos were injected into either both cells at the 2-cell stage or the animal cells at the 4- to 8-cell stage with either mRNA or morpholino. To induce a neural plate border/neural crest state, dissected explants were immediately cultured in 3 µM K02288 (Sigma-Aldrich) and 107 µM CHIR99021 (Sigma-Aldrich) in 1× MMR, as described by Huber and LaBonne (2024) and remained in pharmacological solution until the time of collection. To induce a neural state, dissected explants were immediately cultured in 20 µM K02288 (Sigma-Aldrich) and remained in pharmacological solution until the time of collection.

### Western blotting

Five whole embryos or ten explants were lysed in 1% NP-40 supplemented with protease inhibitors [Complete Mini, EDTA-free tablet (Roche), Leupeptin (Roche), Aprotinin (Sigma-Aldrich) and phenylmethylsulfonyl fluoride (PMSF; Sigma-Aldrich)]. SDS page and western blot were used to detect proteins. The following primary antibodies were used: c-Myc 9E10 (1:3000; Santa Cruz Biotechnology; sc-40); FLAG M2 (1:3000; Sigma-Aldrich; F1804); actin (1:5000; Sigma-Aldrich; A2066); Sox3 (1:200; gift from Dominique Alfandari, University of Massachusetts Amherst, Department of Veterinary and Animal Sciences, Amherst, MA, USA). IRDyes (1:20,000; mouse-800 CW; rabbit-680 TL) and the Odyssey platform (LI-COR Biosciences) were used to detect proteins. Image Studio Lite software was used to quantify protein. Results are representative of a minimum of three biological replicates.

### Co-immunoprecipitation

Five whole embryos were lysed in 1% NP-40 supplemented with protease inhibitors (see above). A 5% input was retained for western blot analysis and the remaining 95% incubated with c-Myc 9E10 antibody (1:500) for 1 h. Approximately 25-30 µl of PAS beads (Sigma-Aldrich; P3391) were added to the lysate and incubated for 2 h. Beads were washed with 1% NP-40 and remaining proteins eluted off the beads. Input and immunoprecipitation samples were analyzed by western blotting as described above. Results are representative of a minimum of three biological replicates.

### RNA isolation, library prep, and sequencing

RNA was extracted from 10-12 explants using Trizol (Life Technologies) followed by LiCl precipitation. Approximately 300 ng of RNA was used for library prep (NEBNext^®^ Ultra™ II Directional RNA Library Prep Kit for Illumina) following standard kit procedure. Libraries were pooled and sequence using NextSeq 500 system (single end, 75 bp). Results are representative of three biological replicates.

### Quantitative PCR

RNA was isolated as described above and converted into cDNA using a High-Capacity cDNA Reverse Transcription Kit (Life Technologies). qPCR was performed using SYBR Premix (Clontech, RR820W) using the primer sequences listed in Table S1. Fold expression was normalized to ornithine decarboxylase (*odc*) and relative to wild type. The ΔΔCT method was used to calculate fold expression and data are represented as a mean from three separate biological replicates with error bars representing standard deviation.

### HCR and immunofluorescence

HCR methodologies were slightly modified from those described by Choi et al. (2018). Whole embryos or explants were hybridized with DNA probe sets for *pax3* (exonic and intronic), *sox10*, *foxd3*, *pou5f3*.*1*, *pou5f3*.*2* (Molecular Instruments) and incubated overnight at 37°C. Probe was removed and samples were washed and then incubated overnight with DNA hairpins labeled with Alexa 647 or Alexa 546 (Molecular Instruments). Unbound hairpins were removed by four 15-minute washes with 5× SSC and then samples were immediately blocked in 10% fetal bovine serum with 0.1% Triton X-100 (in PBS) in preparation for Sox3 immunostaining. Samples were incubated for 1 h at room temperature with Sox3 antibody (1:100; gift from Dominique Alfandari) or c-Myc 9E10 (1:1000), washed, and incubated for 1 h with Alexa 488/568 secondary antibodies [1:1000; Life Technologies, mouse, A11011 (488) and A11004 (568)] and DAPI (1:5000; Life Technologies). Sox2 antibody was used at 1:500 (Cell Signaling Technologies; 3579S). Samples were mounted and imaged using a Nikon C2 upright confocal with two GaAsP detectors and four standard laser lines.

### Colocalization analysis

Maximum intensity projections of explants were made from confocal files using Fiji. Sox3 and *pax3* expression was autothresholded using the RenyiEntropy method, and DAPI was auto local thresholded using the Bernsen method using Fiji. To discern real cells, only connected clusters of 20 DAPI-expressing pixels or larger were selected to be analyzed. DAPI-expressing cells were quantified and used as an overlay to quantify *pax3-* and Sox3-positive cells in those respective channels. The number of *pax3*-postive cells, Sox3-positive cells, and p*ax3*/Sox3 double-positive cells were quantified. Three regions in each explant, with 50 DAPI-positive cells in each region, were randomly selected for further analysis. Within each region, the total number of *pax3-*postive cells that were also Sox3 positive (Sox3&*pax3*^++^/total *pax3*^+^ cells) were quantified. Eighteen regions in total were analyzed per stage (three replicates; two explants per stage; three regions per explant; total sample size: 18). All values were visualized as violin plots (ggplot2) using the Tukey method to define outliers.

### ChIP

Fifty wild-type or Myc-tagged, Sox3-expressing blastula stem cell explants were crosslinked for 15 min with 1% methanol-free formaldehyde (Life Technologies) and the reaction was quenched with 125 mM glycine in 0.5% Triton X-100 in PBS. Explants were washed with 0.01× MMR then lysed using ChIP lysis buffer (5 mM Tris-HCl, pH 7.4, 15 mM NaCl, 1 mM EDTA, 1 mM DTT, 1% NP-40, 0.25% sodium deoxycholate, 0.1% SDS with protease inhibitors). Lysates were briefly sonicated using a M220 focused ultrasonicator and incubated with Myc antibody (1:500; Sigma-Aldrich, C3956) overnight at 4°C. Dynabeads Protein G (Life Technologies; 10004D) were added to the samples and incubated for 1 h. Beads were washed with Tagmentation Wash Buffer (10 mM Tris, pH 7.5, 5 mM MgCl_2_) and incubated with Tn5 transposes (Illumina Tagment DNA Enzyme and Buffer Small Kit) for 40 min at 37°C in a thermomixer (1000 rpm). Tagmented samples were washed, eluted from beads, reverse crosslinked, and purified using a QIAGEN MinElute Reaction Clean Up kit (#28204). DNA was amplified using Nextera primers, adapter dimers removed with AMPure XP beads (Beckman Coulter), and library quality assessed using an Agilent 4150 TapeStation. Pooled libraries were sequenced using NextSeq 500 (blastula) or NovaSeq (neural plate border) systems (paired end, 75 bp). Results are representative of three biological replicates.

### DNA constructs

*Xenopus laevis sox2* and *sox3* clones were obtained from Open Biosystems and subcloned into a pCS2+ vector to add either five N- or C-terminal Myc tags (nMT/cMT). All constructs were verified by sequencing. *Xenopus laevis pou5f3.1/2/3* constructs have been described by York et al. (2023 preprint).

### RNA-seq analysis

Fastq files were obtained from the NuSeq Core facility. Sequences were trimmed using fastp (Chen et al., 2018) and aligned to the *Xenopus laevis* genome (9.2) using STAR or RSEM [for transcripts per million (TPMs)] (Dobin et al., 2013; Li and Dewey, 2011). TPM data is presented as an average of three biological replicates of combined data from S and L alleles for each gene, with the width of each line representing standard deviation. HTseq was used to generate read counts from STAR aligned bam files (Putri et al., 2022). Differential expression analysis was performed using DESeq2 with significance defined as *P*_adj_≤0.05 (Love et al., 2014). Volcano plots were generated using ggplot2 (Wickham, 2016). Fastq files from Plouhinec et al. (2017) were retrieved from Gene Expression Omnibus (GSE103240) and TPMs were generated using the above methods. Anterior neural border samples for stages 12.5 and 14 were used along with stage 17 neural crest samples.

### ChIP-seq analysis

Fastq files were obtained from the NuSeq Core facility. Sequences were trimmed using fastp (Chen et al., 2018) and aligned to the *Xenopus laevis* genome (9.2) using bowtie2 (Langmead and Salzberg, 2012). Samtools was used to process files (Danecek et al., 2021). Name sorted bam files were used for peak calling using genrich (-t Sox3 bam files; -c wildtype bam files; -v; -e MT; -a 20) (available at https://github.com/jsh58/Genrich). HOMER was used to annotate peaks and for motif analysis (Heinz et al., 2010). The FIMO tool from MEME Suite was also used for motif analysis (Grant et al., 2011). The GO Consortium was used for GO term analysis, using the *Homo sapiens* database (Ashburner et al., 2000; Gene Ontology Consortium, 2021). IGV was used for browser track visualization (Robinson et al., 2011).

### Statistics

Fisher's exact test was performed on 2×2 contingency plots and odds ratios visualized by forest plots generated in ggplot2. All other statistical analyses used two-tailed *t*-tests to determine significance.

## Supplementary Material

10.1242/develop.202693_sup1 Supplementary information
