# Supplementary material for: SoxB1 transcription factors are essential for initiating and maintaining neural plate border gene expression
Source: Development. 2024 Jul 22;151(14):dev202693. doi: 10.1242/dev.202693 (PMC11369808; doi:10.1242/dev.202693)
Supplement: Supplementary information [file develop-151-202693-s1.pdf]

Sup.Fig. 1

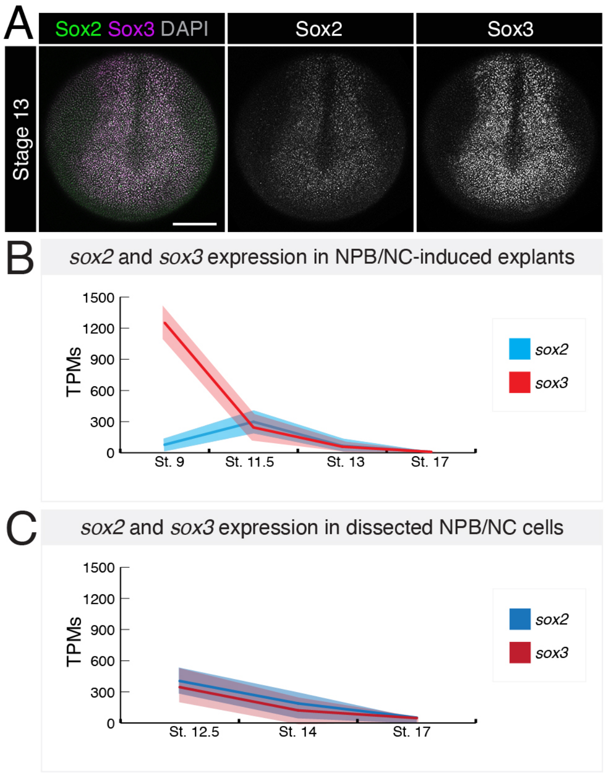

Fig. S1. Sox2 and Sox3 have overlapping expression domains. (A) Wildtype stage 13 embryo immunostained for Sox2 (green) and Sox3 (magenta). DAPI is shown in gray. (B) Average TPMs for *sox2* (blue) and *sox3* (red) in blastula stem cells (St. 9) and neural plate border/neural crest-induced explants (St. 11.5, St. 13, St. 17). (C) Average TPMs for *sox2* (blue) and *sox3* (red) from dissected neural plate border/neural crest cells (St. 12.5, St.14, St.17). Error bars are standard deviation. Neural plate border (NPB); neural crest (NC). Scale bar: 250 µm.

Development • Supplementary information

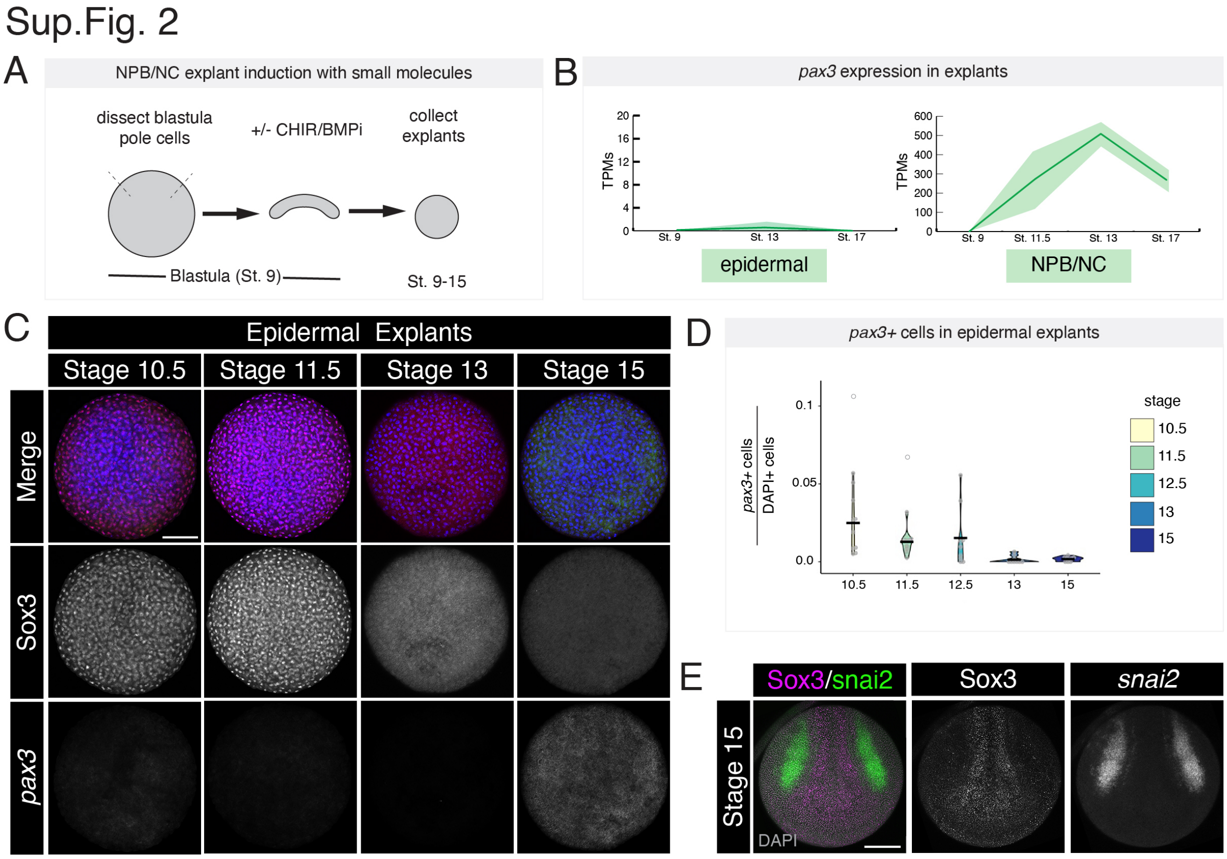

Fig. S2. Temporal relationship between Sox3 and neural plate border gene expression. (A) Experimental set up for neural plate border/neural crest induction of blastula stem cell explants using small molecules. (B) Average TPMs for *pax3* in epidermal vs neural crest-induced explants (St. 9, 11.5, St. 13, St. 17). Error bars are standard deviation. (C) Nascent *pax3* expression (green) and *Sox3* protein (red) in epidermal (control) explants from early gastrulation through mid-neurulation. DAPI is shown in blue. (D) Quantification of percent *pax3*+ cells in epidermal explants. (E) Wildtype stage 15 embryo immunostained for Sox3 (magenta) and probed for *snai2* (green) using HCR. DAPI is shown in gray. Neural plate border (NPB); neural crest (NC). Scale bars: 125 µm (C); 250 µm (E).

Development • Supplementary information

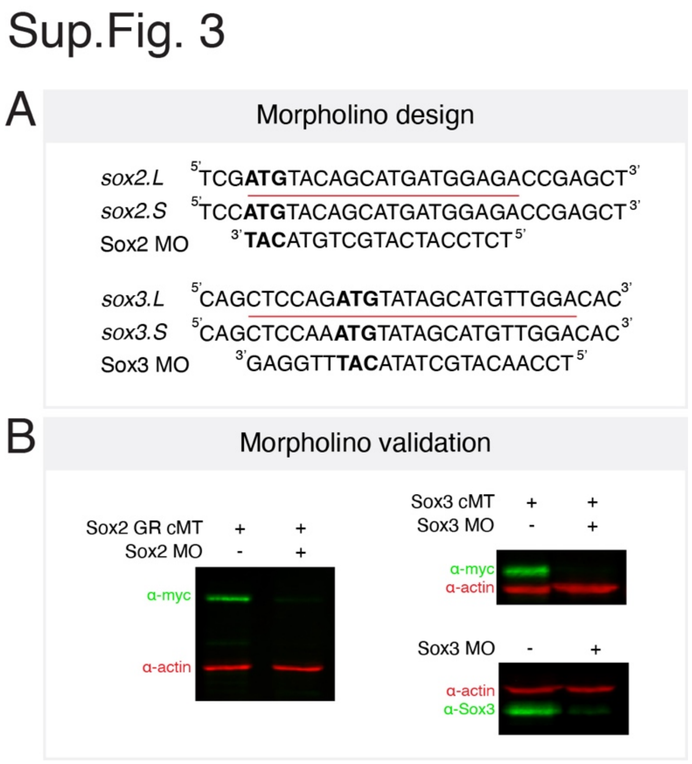

Fig. S3. Sox2 and Sox3 morpholino validation. (A) Schematic showing morpholino sequences and target regions at *sox2* and *sox3* alloalleles. (B) Western blot validation of Sox2 and Sox3 morpholinos. C-terminal myc tag (cMT); glucocorticoid receptor (GR); morpholino (MO).

Development • Supplementary information

Sup.Fig. 4

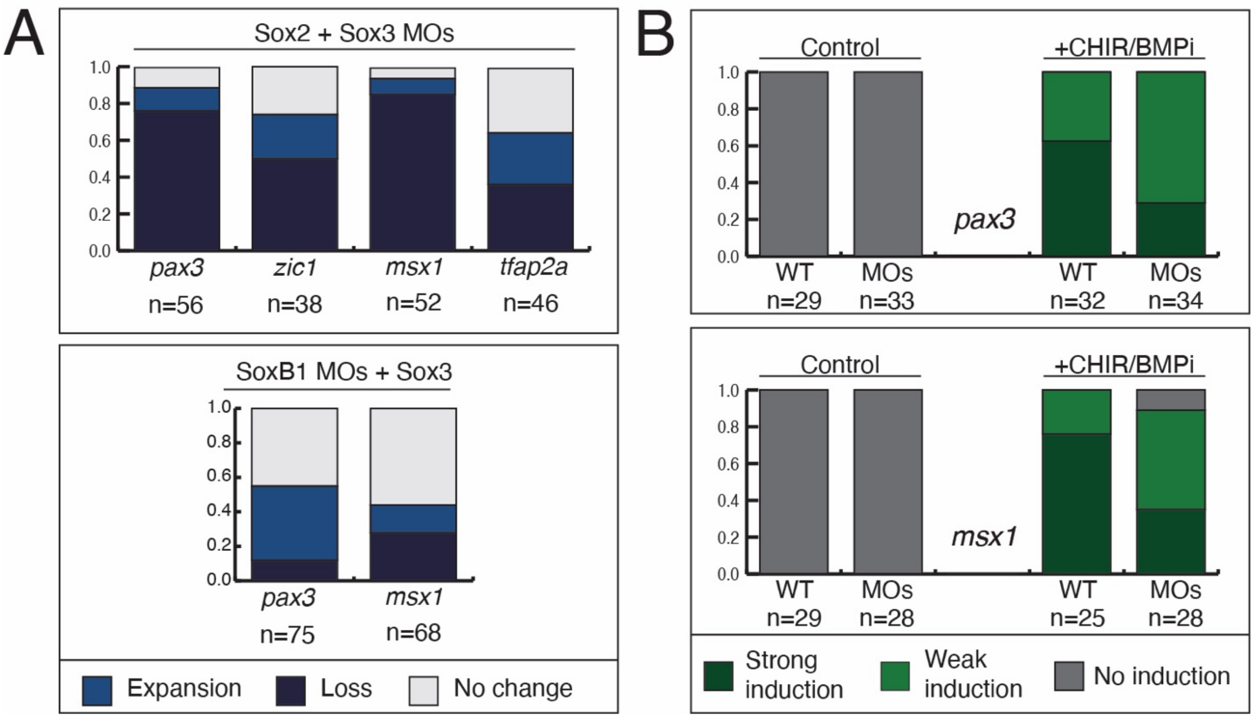

Fig. S4. *soxb1* morphant scoring. (A) Stacked bar graphs with the percent of embryos with changes in gene expression (loss, expansion, no change) for *sox2* and *sox3* double morphants and rescued morphants (B) Stacked bar graphs with the percent of neural plate border-induced explants expressing *pax3* or *msx1*, indicating induction to a neural plate border state. Wildtype (WT); morpholino (MO).

Development • Supplementary information

Sup.Fig. 5

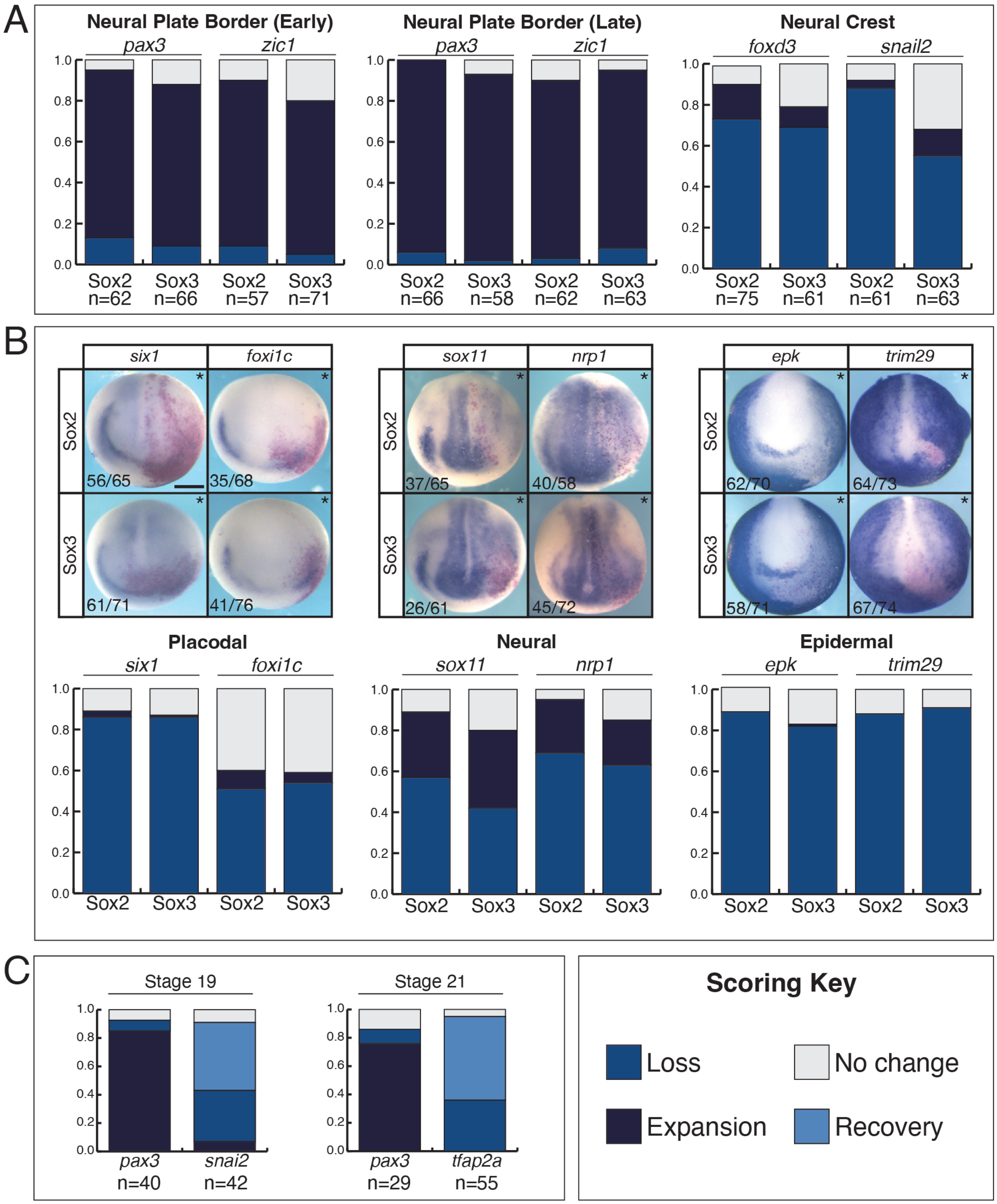

Fig. S5. *In situ* hybridization and scoring for Sox2 and Sox3 expressing embryos. (A) Stacked bar graphs with the percent of embryos with changes in gene expression (loss, expansion, no change) in *sox2* or *sox3* expressing embryos. (B) *In situ*

Development • Supplementary information

hybridization in stage 16 embryos unilaterally expressing *sox2* or *sox3* mRNA (* denotes injected side) probing for placodal markers (*six1* and *foxi1c),* neural markers (*sox11* and *nrp1)*, and epidermal markers (*epk* and *trim29*) with associated scoring. Beta-galactosidase (red) was used as a lineage tracer. Stacked bar graphs with the percent of embryos with changes in gene expression (loss, expansion, no change) in *sox2* or *sox3* expressing embryos. (C) Stacked bar graphs with the percent of *sox3* expressing embryos with changes in gene expression (loss, expansion, recovery, no change) at stage 19 and 21. Scale bar: 250 µm.

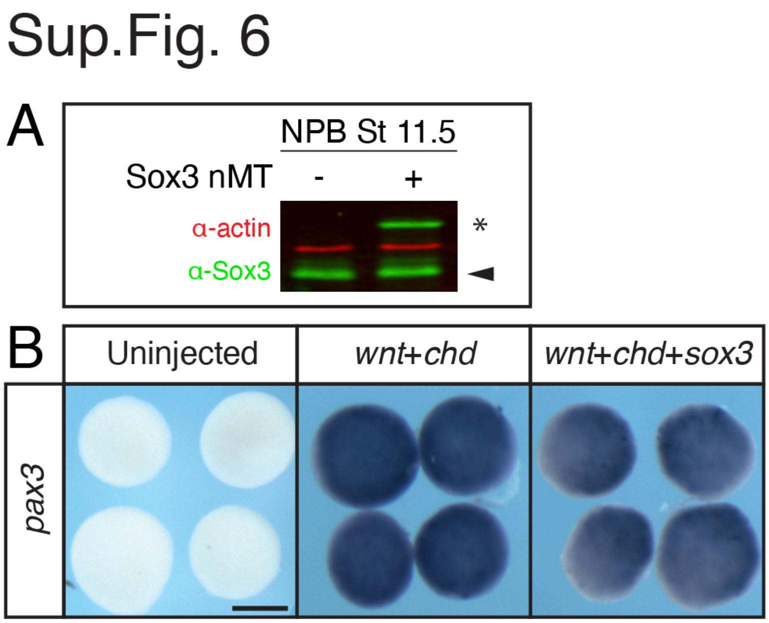

Fig. S6. Neural plate border induction assessment for ChIP-seq experiments. (A) Western blot for Sox3 (green) and actin (red) in wildtype and *sox3* injected neural plate border-induced explants (St. 11.5). Arrowhead denotes endogenous protein and * deonotes myc-tagged sox3. (B) *In situ hybridization* for *pax3* in neural plate border-induced explants with and without myc-tagged sox3. Scale bar: 250 µm.

Development • Supplementary information

Sup.Fig. 7

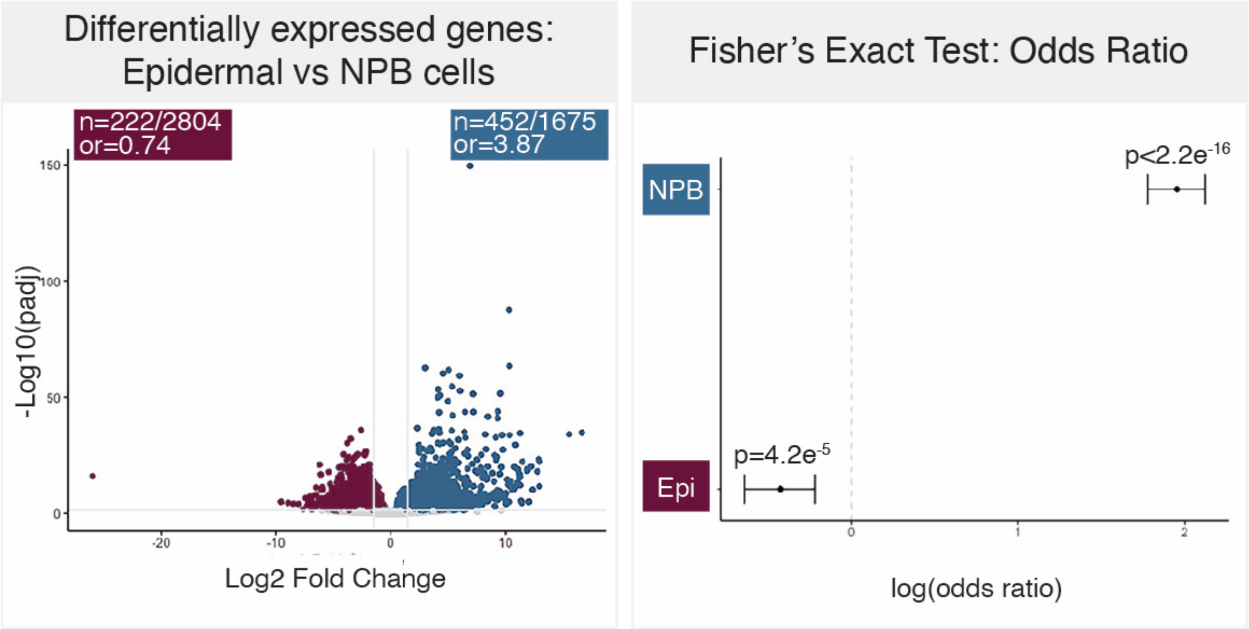

Fig. S7. Fisher's exact tests on differentially expressed genes in relation to Sox3 binding. Volcano plot showing differentially expressed genes between stage 13 epidermal and neural plate border-induced explants. Genes differentially expressed in epidermal cells are shown in maroon and in blue for neural plate border cells. Forrest plot displaying the log(odds ratio) for each group of differentially expressed genes (epidermal vs neural plate border) in relation to Sox3 binding in neural plate border-induced explants (St. 11.5). Odds ratio (or); neural plate border (NPB), epidermis (Epi).

Sup. Fig. 8

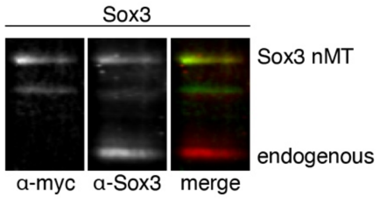

Fig. S8. Sox3 was expressed at near endogenous levels for blastula (St. 9) ChIP-seq experiments. Western blot for myc (green) and Sox3 (red) in embryos expressing *sox3* mRNA.

Development • Supplementary information

Sup.Fig. 9

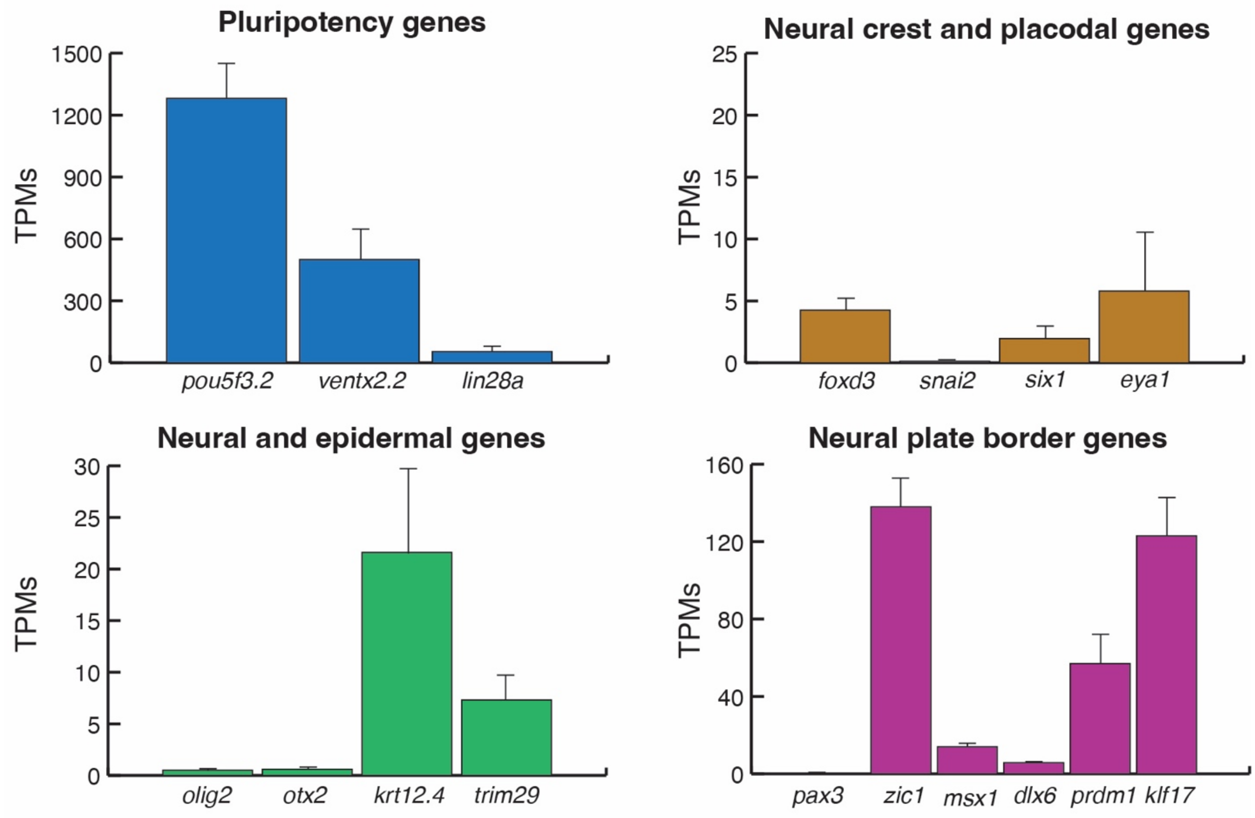

Fig. S9. TPMs for genes in blastula stems cells. Average TPMs for pluripotency genes (*pou5f3.2, ventx2.2* and *lin28a*), neural crest genes (*foxd3* and *snai2*), placodal genes (*six1* and *eya1*), neural genes (*olig2, otx2),* epidermal genes (*krt12.4, trim29*), and neural plate border genes (*pax3, zic1, msx1, dlx6, prdm1,* and *klf17*) in blastula stem cells (stage 9). Error bars are standard deviation.

Sup. Fig. 10

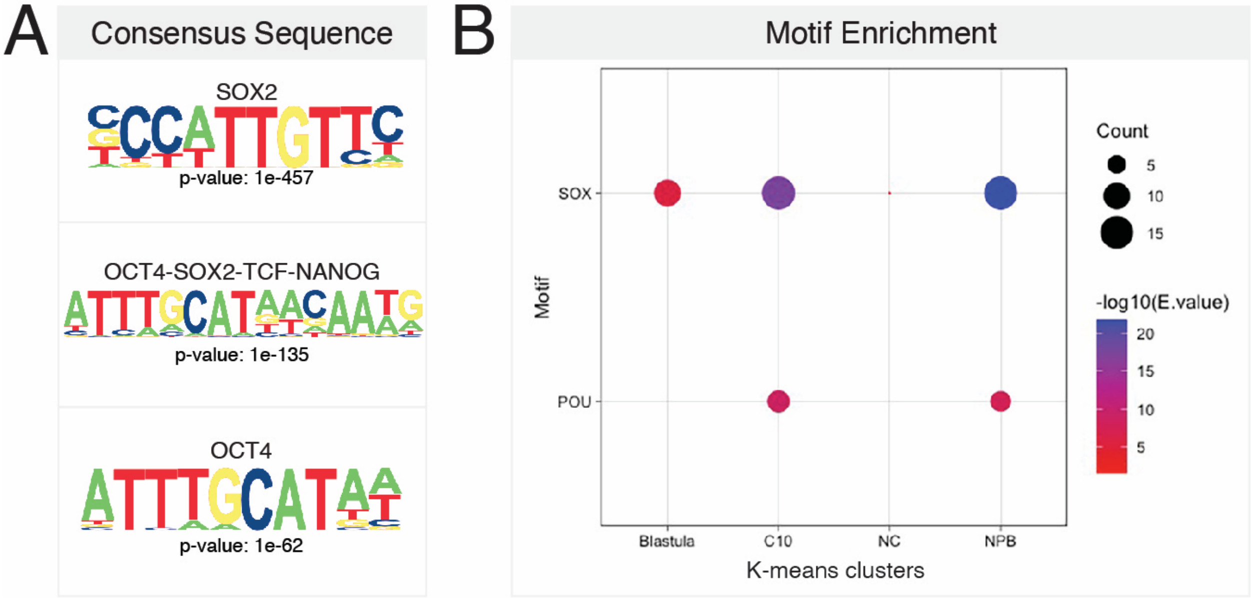

Fig. S10. Motif analysis on shared Sox3 ChIP-seq peaks (St.9 and St. 11.5). (A) HOMER motif consensus sequences and associated p-value. (B) Motif enrichment analysis, focusing on prevalence of SOX and POU motifs, on regions of Sox3 binding in the four k-means clusters.

Sup.Fig. 11

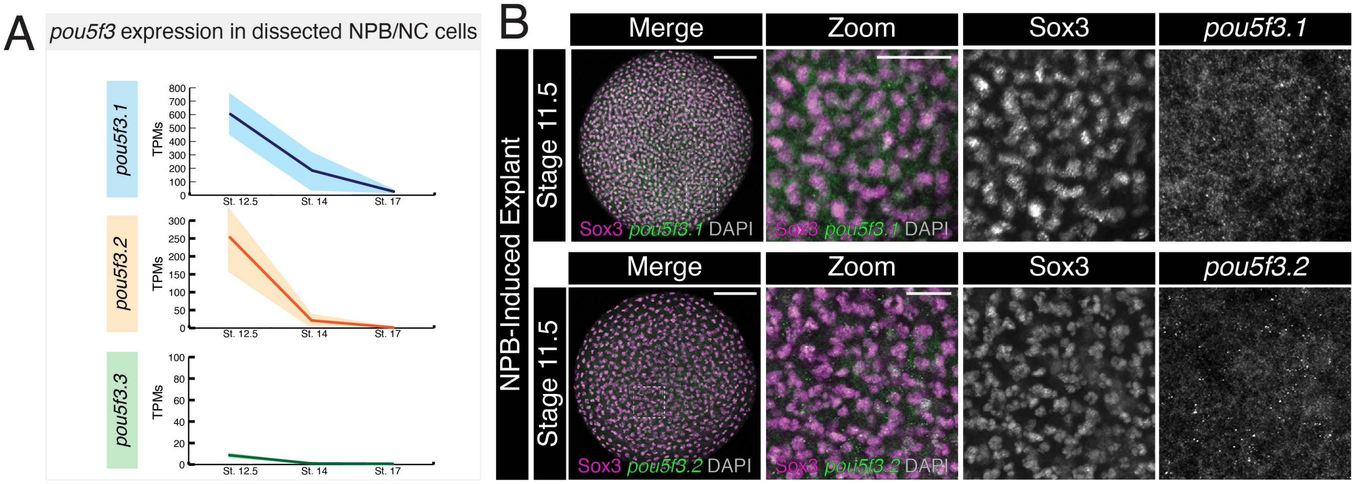

Fig. S11*. pou5f3.1/2* are expressed in neural plate border cells (A) Average TPMs for *pou5f3.1* (blue), *pou5f3.2* (orange), and *pou5f3.3* (green) from dissected neural plate border/neural crest cells (St. 12.5, St.14, St.17). Error bars are standard deviation. (B) Neural plate border-induced explants (stage 11.5) immunostained for Sox3 (magenta) and probed with HCR oligos for *pou5f3.1* or *pou5f3.2* (green). DAPI is shown in gray. Neural plate border (NPB); neural crest (NC). Scale bars: 125 µm (B); 50 µm (B, zoom).

Development • Supplementary information

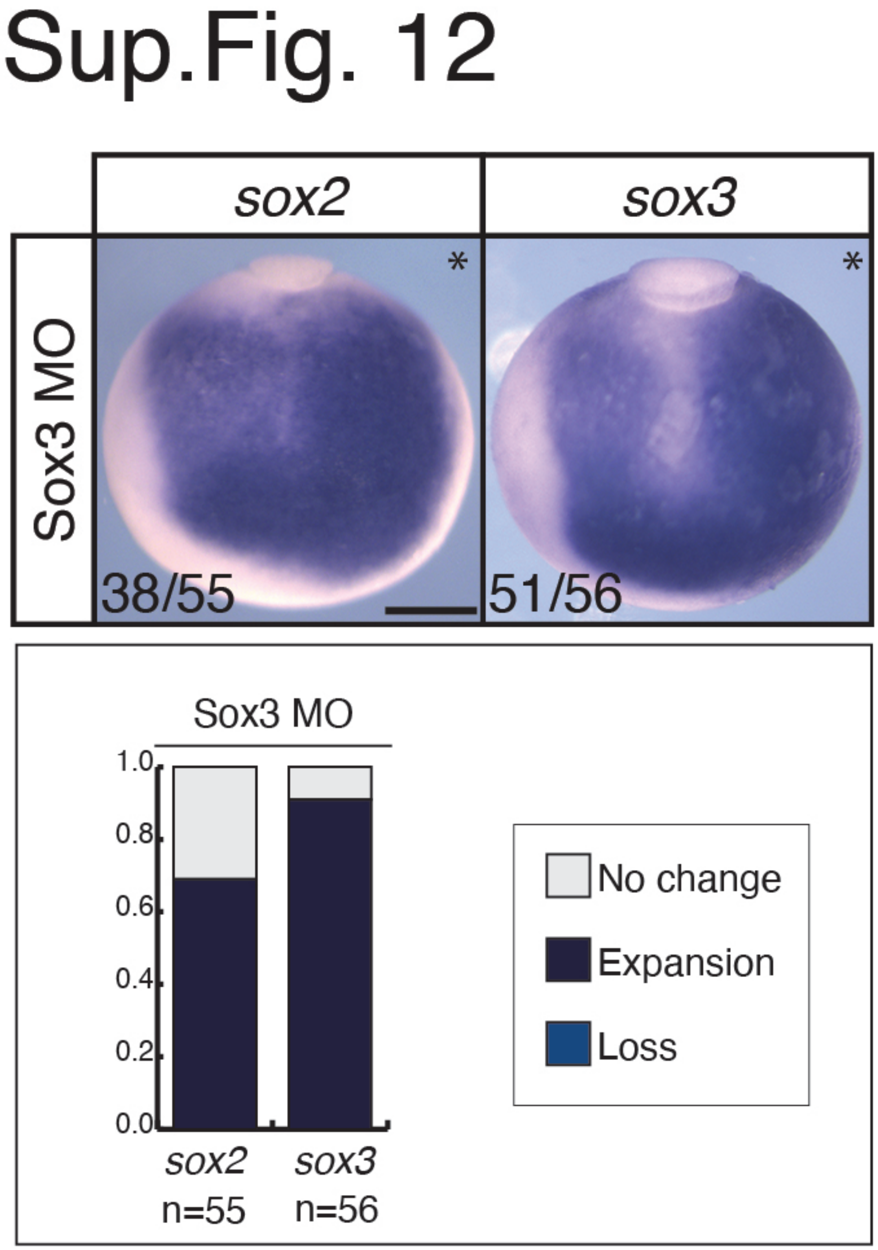

Fig. S12. *sox3* morphants have expanded *sox2* and *sox3* expression. *In situ hybridization* for *sox2* and *sox3* in stage 12.5 *sox3* morphant embryos (* denotes injected side). Fluorescein dextran was used as a lineage tracer and embryos were presorted for left/right side targeting. Stacked bar graphs with the percent of embryos with changes in gene expression (loss, expansion, no change) for *sox3* morphants. Morpholino (MO). Scale bar: 250 μm.

Development • Supplementary information

Sup.Fig. 13

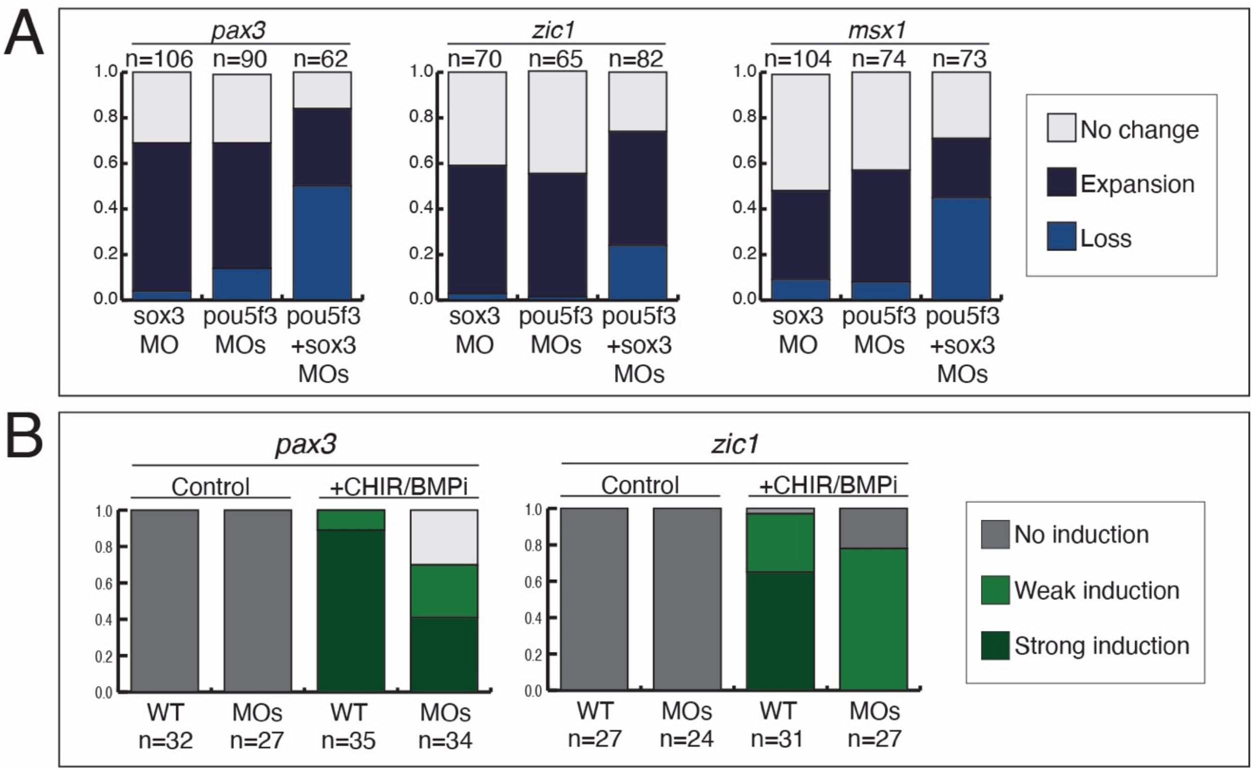

Fig. S13. *Pou5f3.1/2* + *sox3* triple morphant scoring. (A) Stacked bar graphs with the percent of embryos with changes in gene expression (loss, expansion, no change) in *sox3* morphants, *pou5f3.1+pou5f3.2* double morphants, and sox3+*pou5f3.1+pou5f3.2* triple morphants (B) Stacked bar graphs with the percent of neural plate border-induced explants expressing *pax3* or *zic1*, indicating induction to a neural plate border state. Wildtype (WT); morpholino (MO).

Table S1. qPCR primer sequences

Gene	Forward Primer	Reverse Primer
odc	TGAAAACATGGGTGCCTACA	TGCCAGTGTGGTCTTGACAT
pax3	AGGGGCTTTTCCTCCTACAG	TTGCTTGGATCAGACACAGC
zic1	CCTGGATGTGGCAAAGTCTT	GTCACAGCCTTCAAACTCGC
msx1	CCCAACAAGAAGGATGAGCC	TCTCCAGAGCCAGCAGTTG
dlx5	GCGCTGAATGCGTATCAGTA	AGGGCTCCCATAGCCATAGT
prdm1	CCTTAAGGTGCATCTTCGGG	TGCTTCTGTAAATGCGCCAG
klf17	AGTGTGGCTTTCTCAACCCT	TATCAGCCAGTGGTCTCAGC

Development • Supplementary information
